# Sequencing and culture-based characterization of the vaginal and uterine microbiota in beef cattle that became pregnant or remained open following artificial insemination

**DOI:** 10.1128/spectrum.02732-23

**Published:** 2023-11-03

**Authors:** Emily M. Webb, Devin B. Holman, Kaycie N. Schmidt, Beena Pun, Kevin K. Sedivec, Jennifer L. Hurlbert, Kerri A. Bochantin, Alison K. Ward, Carl R. Dahlen, Samat Amat

**Affiliations:** 1 Department of Microbiological Sciences, North Dakota State University, Fargo, North Dakota, USA; 2 Lacombe Research and Development Centre, Agriculture and Agri-Food Canada, Lacombe, Alberta, Canada; 3 Central Grasslands Research Extension Center, North Dakota State University, Streeter, North Dakota, USA; 4 Department of Animal Sciences and Center for Nutrition and Pregnancy, North Dakota State University, Fargo, North Dakota, USA; Texas A&M University, College Station, Texas, USA

**Keywords:** vaginal microbiota, uterine microbiota, bovine, 16S rRNA gene sequencing, culturing, pregnancy, artificial insemination, antimicrobial resistance

## Abstract

In this study, we evaluated the vaginal and uterine microbiota between beef cattle that became pregnant via artificial insemination (AI) and those that remained open to identify microbial signatures associated with fertility. We also characterized the culturable fraction of these microbiota and screened certain vaginal and uterine bacterial isolates for antimicrobial resistance. For this, vaginal and uterine swabs were collected before AI from two cohorts of Angus-crossbred cattle: mature cows (vaginal and uterine: 27 open and 31 pregnant) and heifers (vaginal: 26 open and 33 pregnant). The microbiota of these samples were assessed using 16S rRNA gene sequencing and culturing. Twenty-nine vaginal and uterine bacterial isolates were screened for resistance against 41 antibiotics. Within the vaginal microbiota, 11 amplicon sequence variants (ASVs) were more relatively abundant in the open heifers compared with the pregnant heifers. No differentially abundant ASVs were detected in the vaginal samples from pregnant versus open cows. Pregnant cows had a distinct uterine microbiota community structure (*P* = 0.008) and interaction network structure compared with open cows. Twenty-eight differentially abundant uterine ASVs were observed between the pregnant and open cows. Community structure and diversity were different between the cow’s vagina and uterus. A total of 733 bacterial isolates were recovered from vaginal (512) and uterine (221) swabs under aerobic (83 different species) and anaerobic (69 species) culturing. Among these isolates were pathogenic species and those mostly susceptible to tested antibiotics. Overall, our results indicate that fertility-associated taxonomic signatures are present in the bovine uterine and vaginal microbiota.

**IMPORTANCE:**

Emerging evidence suggests that microbiome-targeted approaches may provide a novel opportunity to reduce the incidence of reproductive failures in cattle. To develop such microbiome-based strategies, one of the first logical steps is to identify reproductive microbiome features related to fertility and to isolate the fertility-associated microbial species for developing a future bacterial consortium that could be administered before breeding to enhance pregnancy outcomes. Here, we characterized the vaginal and uterine microbiota in beef cattle that became pregnant or remained open via artificial insemination and identified microbiota features associated with fertility. We compared similarities between vaginal and uterine microbiota and between heifers and cows. Using culturing, we provided new insights into the culturable fraction of the vaginal and uterine microbiota and their antimicrobial resistance. Overall, our findings will serve as an important basis for future research aimed at harnessing the vaginal and uterine microbiome for improved cattle fertility.

## INTRODUCTION

Reproductive failure is a major factor in the beef and dairy cattle industry, resulting in significant and negative management and economic impacts despite recent advances in artificial insemination (AI), genetic selection, and improved nutrition and management of cattle over the past several decades (1 – 3). A recent meta-analysis stated that nearly 48% of beef cows experience loss of pregnancy within the first 30 days of gestation after a single insemination, and roughly 6% of pregnancy loss occurs during the remaining gestation period (3). Therefore, improving fertility and reducing pregnancy loss in beef cattle are very important in maintaining sustainable beef production.

It is increasingly known that the microbiota residing within the female reproductive tract of humans are important for reproductive health and fertility (4 – 7). While causality still remains to be determined, associations of the vaginal and uterine microbiota with fertility, implantation, and preterm birth have been relatively well documented (5, 8 – 11). In bovine animals, the taxonomic composition of the vaginal and uterine microbial communities and factors shaping these communities as well as their association with reproductive health have increasingly been explored (12 – 15). Galvão and colleagues identified the association between the microbiome and uterine disease in dairy cows. The cows with metritis exhibited dysbiosis of the uterine microbiota, which was characterized by reduced species richness and an increase in the relative abundance of the phyla Bacteroidota and Fusobacteriota (16). Likewise, the uterine microbiota of dairy cows diagnosed with clinical endometritis (CE) differed significantly from that of healthy cows and was characterized by reduced microbial diversity and an enrichment of *Trueperella pyogenes* (17). Besides the involvement of the uterine microbiome in uterine health, recent evidence suggests that dysbiosis of this microbiota prior to breeding may compromise host fertility (18 – 20). Thus, given the evidence from both humans and cattle suggesting a role for the vagino-uterine microbiomes in reproductive health and fertility, we hypothesize that fertility- related taxonomic signature may be present in the bovine vaginal and uterine microbiota. The present study was conducted to evaluate the following objectives: (i) characterize the vaginal and uterine microbiota of virgin yearling heifers (with no pervious breeding history with bulls or AI) and cows (primiparous and multiparous) at the time of AI breeding, (ii) identify differentially abundant taxa between cattle that became pregnant and those that remained open following AI, (iii) characterize the culturable fraction of the vaginal and uterine microbiota, and (iv) investigate antimicrobial resistance (AMR) in these vaginal and uterine bacterial isolates. Of note, in addition to sequencing, we applied culturing to provide higher taxonomic resolution and isolate and bank culturable bacterial species that could be used for the development of microbiome-targeted strategies to improve female fertility in cattle. We also evaluated AMR in these bacterial isolates given that the female reproductive tract could serve a medium for transferring antimicrobial-resistant bacteria from mother to offspring and from female to bulls and then from bulls to other females.

## MATERIALS AND METHODS

All experimental procedures involving cattle were approved by the North Dakota State University Institutional Animal Care and Use Committee (IACUC protocol# A21061 and IACUC# 21049 for cows and heifers, respectively).

### Animal husbandry and experimental design

Two cohorts of Angus-based cross-bred female cattle: cows (*N* = 100) and heifers (*N* = 72), were selected for collection of vaginal (cows and heifers) and uterine (cows only) swabs 2 days prior (heifers) or at the time of AI (cows). Cows and heifers were estrus synchronized using a 7-day Co-Synch + controlled internal drug release device [Eazi-Breed CIDR (controlled internal drug release dispenser) progesterone intravaginal insert, Zoetis, Parsippany, NJ, USA] for fixed-time AI (21). Briefly, cattle were intramuscularly administered 2 mL of gonadotropin-releasing hormone as gonadorelin injection (GnRH; Factrel Injection, Zoetis, Parsippany, NJ, USA) on day 0 of the estrus synchronization protocol. Upon wiping the vulva clean, the CIDR device was inserted into the vagina on day 0 and remained implanted until day 7. On day 7 of the protocol, the CIDR was removed and females were intramuscularly administered 2 mL of prostaglandin (Lutalyse HighCon, Zoetis, Parsippany, NJ, USA) to stimulate estrus. Cows were bred via fixed-time AI at approximately 60 to 66 h after CIDR removal and given an additional 2 mL dose of GnRH at the time of AI. Heifers were bred with female-sexed semen 16 to 20 h after expression of standing estrus. Non-responding heifers were bred 72 ± 2 h after CIDR removal and were also given an additional dose of GnRH at AI.

Pregnancy diagnosis via ultrasound was performed 35 days after AI. Cows had free access to grazing pastures beginning 45 days before collection. Pasture description and species have been described previously (22). Cows were all postpartum suckled/lactating cows. They were 84 days postpartum at the time of breeding. There is no incidence of postpartum disease.

Heifers were managed on a diet containing alfalfa/grass hay (60% of diet, dry matter basis), ground corn (31%), and corn silage (9%). These heifers were sourced from the same farm where the cows were raised, but, at the time of sample collection, they were raised in the Animal Nutrition and Physiology Center (ANPC) at North Dakota State University (Fargo, ND, USA) and were individually fed using an electronic head-gate facility (American Calan; Northwood, NH, USA)

### Vaginal and uterine swab collection

#### Vaginal swab collection

Vaginal swabs were collected from cows (*N* = 100) at the time of AI and heifers (*N* = 72) 2 days before AI using the method described previously (23). For vaginal swab collection, the vulva was thoroughly cleaned with 70% ethanol and a paper towel. The labia majora was then held open allowing the passage of a swab into the vagina (15 cm, sterile cotton-tipped applicator with aerated tip protector; Puritan, Guilford, ME, USA). When the swab tip reached the midpoint of the vaginal cavity, it was swirled four times, making consistent contact with the vaginal wall, and then carefully withdrawn to minimize contamination. The swabs were immediately placed into sterile Whirl-Pak bags (Uline, Pleasant Prairie, WI, USA) placed on ice and transported to the lab.

#### Uterine swab collection

Uterine samples were only collected from cows as an acceptable uterine swabbing technique for virgin yearling heifers was not available at the time of sampling. Immediately after vaginal swab collection from each cow, uterine swab sampling was performed using a double-guarded culture swab (71 cm in length, Reproduction Provisions L.L.C., Walworth, WI, USA). The double-guarded culture swab was guided via rectal palpation through the vagina to the cervix; then, the inner plastic portion was threaded through the cervix and into the uterine body. Once in the uterine body, the swab tip was extended through the inner plastic sheath. Gentle pressure was applied by pinching the uterine body to the swab via rectal palpation, and the swab was then rotated three times. Once the sample was collected, the swab was retracted into the inner and outer plastic sheaths and removed from the cow. The tip of the swab was then cut and placed in a 2-mL tube, placed on ice, and transported to the lab. Of note, the vaginal and uterine swabs were collected simultaneously from all of the cows prior to AI and within a 4-h period by the same personnel.

At the lab, the uterine and vaginal swabs were transferred into 1 mL of brain heart infusion (BHI) broth (Hardy Diagnostics, Santa Maria, CA, USA) containing 20% glycerol (Fisher Scientific, Fair Lawn, NJ, USA) and stored at −80°C until genomic DNA extraction and culturing.

### Metagenomic DNA extraction

The DNeasy Blood and Tissue Kit (Qiagen Inc., Hilden, Germany) was used to extract genomic DNA from the vaginal and uterine swab samples. Prior to extraction, the samples were thawed and then thoroughly vortexed before transferring 500 µL of the BHI + 20% glycerol containing a swab to a new 2-mL screw cap tube. This tube was then centrifuged at 20,000 × *g* for 10 min at 4°C, and the supernatant was carefully discarded to avoid disturbing the pellet. Using a sterile scalpel and/or forceps, the cotton tip was removed from the swab handle and added to the corresponding cell pellet. Next, 350 µL of enzymatic lysis buffer [20 mM Tris.Cl (pH 8), 2 mM sodium ethylenediaminetetraacetic acid (EDTA), 1.2% Triton X-100, lysozyme (100 mg/mL), and mutanolysin (25,000 U/mL)] was added to each tube before vortexing to dissolve the pellet and submerge the cotton. The tubes were then incubated for 1 h at 37°C with agitation at 800 rpm (23). The DNeasy Blood and Tissue Kit was then used as described in the manufacturer’s protocol with the addition of two rounds of bead beating at 6.0 m/s for 40 s using a FastPrep-24 Bead Beater (MP Biomedicals, Irvine, CA, USA) after the proteinase K incubation. The DNA was eluted with 50 µL of pre-warmed elution buffer. A negative extraction control was also included. The quantity and quality of the extracted DNA were measured using a NanoDrop ND-1000 spectrophotometer and PicoGreen assay. The DNA was stored at −20°C until 16S rRNA gene library preparation and sequencing.

### 16S rRNA gene sequencing and analysis

The V3-V4 hypervariable regions of the 16S rRNA gene were amplified as described previously (23, 24). Briefly, Phusion High-Fidelity PCR Master Mix (New England Biolabs, Ipswich, MA, USA) was used for all PCR steps and the DNA fragment of interest was excised from a 2% agarose gel and purified with a QIAquick Gel Extraction Kit (Qiagen Inc.). The NEBNext Ultra DNA Library Prep Kit (New England Biolabs) for Illumina was used for sequencing library preparation following the manufacturer’s recommendations. A Qubit 2.0 Fluorometer (Thermo Scientific, Waltham, MA, USA) and Agilent Bioanalyzer 2100 system were used to assess library quality. The 16S rRNA gene libraries were then sequenced on a NovaSeq 6000 instrument with a SP flow cell (2 × 250 bp) (Illumina Inc., San Diego, CA, USA).

The DADA2 v. 1.18 package (25) in R. 4.0.3 was used to process the 16S rRNA gene sequences. The forward reads were truncated at 225 bp and the reverse reads at 220 bp. The reads were merged, chimeric sequences removed, and taxonomy assigned to each merged sequence [amplicon sequence variant (ASV)], using the naïve Bayesian RDP classifier (26) and the SILVA SSU database release 138.1 (27). ASVs were considered to be likely contaminants and removed if they were more abundant in the negative controls on average than within the uterine and vaginal samples. These negative controls included DNA extraction controls as well as unused swabs left open in the room/farm where sample collection took place. ASVs classified as chloroplasts, mitochondria, or eukaryotic in origin were also removed prior to analysis. Phyloseq 1.34.0 (28) and vegan 2.5-7 (29) were used to calculate the number of ASVs per sample (richness), the Shannon and inverse Simpson’ diversity indices, and Bray-Curtis dissimilarities in R. To account for uneven sequence depths, samples were randomly subsampled (without replacement) to 14,500 sequencing reads for the vaginal and uterine samples, prior to the calculation of alpha diversity metrics and Bray-Curtis dissimilarities.

### Isolation of bacteria from vaginal and uterine swab samples

#### Source of swabs used for culturing

Swabs collected from a 2021 cohort of cows (both vaginal and uterine swabs) and heifers (vaginal only) that were used for 16S rRNA gene sequencing were also processed for culturing. In addition, bacteria from vaginal (*n* = 12) and uterine (*n* = 30) swabs collected from a similar cohort of cows and heifers in the following year (2022) were also cultured to increase the diversity of bacterial species isolated from the female reproductive tract. Cryopreserved vaginal and uterine swabs were subjected to aerobic and anaerobic culturing using different growth media as described below.

#### Aerobic culturing

A 50-µL aliquot of undiluted BHI + 20% glycerol stock from uterine and vaginal swab samples was spread onto De Man, Rogosa, and Sharpe (MRS) agar (Hardy Diagnostics, Santa Maria, CA) and Columbia blood agar plates supplemented with 5% sheep’s blood (CB) (Becton, Dickinson and Company, Sparks, MD, USA). Similarly, 50 µL of undiluted vaginal sample was spread on MRS agar, while 50 µL of vaginal sample diluted 1:10 in phosphate buffered saline (PBS) (MediaTech Inc., Manassas, VA, USA) was spread on CB agar. The CB agar plates were incubated at 37°C in 5% CO_2_ and the MRS agar plates at 37°C in 10% CO_2_ for up to 48 h. Up to eight colonies with distinctive morphologies on each plate were then subcultured onto its respective agar and incubated under the same conditions as above. After visually assessing the purity of each isolate, a disposable 100-µL loop was used to transfer each isolate into 100 µL Tris-EDTA (TE) (Quality Biological Inc., Gaithersburg, MD, USA) stock and 1 mL of 20% (vol/vol) glycerol containing MRS or BHI broth depending on what agar the isolate was grown on. The TE stocks were stored at −20°C for genomic DNA extraction, while the MRS (MRSg) and BHI glycerol (BHIg) stocks were stored at −80°C.

#### Anaerobic culturing

Anaerobic culturing was performed in an anaerobic chamber (Type B, Vinyl, Coy Laboratory Products Inc., Grass Lake, MI, USA) supplied with a gas mixture containing 90% N_2_, 5% CO_2_, and 5% H_2_. A subset of vaginal and uterine samples from cows was diluted 1:2 with PBS, and 100 µL was plated onto MRS agar while 50 µL was plated on blood and Wilkins-Chalgren (WC) agar (HiMedia Laboratories, Mumbai, India). These agar plates were incubated at 37°C for up to 72 h. Colonies with a unique morphology were selected and subcultured onto a CB agar plate and incubated at 37°C for up to 72 h. A loopful of each isolate was transferred to 100 µL TE and 1 mL BHI glycerol stock and then stored at −20°C or −80°C, respectively, until used for DNA extraction or culturing.

### Identification of uterine and vaginal isolates

Bacterial strains were identified using amplification and sequencing of the near-full-length 16S rRNA gene. For this, genomic DNA was extracted from all MRS (aerobic: *n* = 122; anaerobic *n* = 104), CB (*n* = 241), blood (*n* = 135), and WC (anaerobic: *n* = 131) isolates using the Quick-DNA Fungal/Bacterial Miniprep Kit (Zymo Research, Irvine, CA, USA) according to manufacturer’s instructions with the modifications outlined in our previous publication (30).

The near-full length 16S rRNA gene was amplified via PCR using the universal primers 27F (5′- AGAGTTTGATCMTGGCTCAG −3′) and 1492R (5′-

TACGGYTACCTTGTTACGACTT −3′) as described previously (30, 31). Each PCR consisted of 20 µL iQ Supermix (Bio-Rad Laboratories Inc., Hercules, CA, USA), 1 µL of each primer (10 µM), and 2 µL of isolate DNA for a total volume of 40 µL per reaction. The PCR conditions were as follows: an initial denaturation of 95°C for 5 min; 35 cycles of 95°C for 45 s, 50°C for 30 s, 72°C for 2 min; and a final extension at 72°C for 5 min, in an Eppendorf Mastercycler (Eppendorf, Hamburg, Germany). A 1% (wt/vol) agarose gel was used to visualize the PCR products, and amplicons were sent to MCLAB (San Francisco, CA, USA) for Sanger sequencing. The 16S rRNA gene sequences were identified using the Basic Local Alignment Search Tool (BLAST) and the non-redundant NCBI nucleotide database.

### Evaluation of antimicrobial susceptibilities of selected vaginal and uterine isolates

To gain insight into AMR in commensal bacteria residing within the female bovine reproductive tract, 29 bacterial isolates (10 Gram-positive and 19 Gram-negative) were selected for antimicrobial susceptibility testing (AST). The selection criteria for inclusion were based on the isolation frequency from the vaginal and uterine swabs, the ability to culture the isolate aerobically as required by the AST method, and most importantly, the availability of the antibiotic breakpoints. Antimicrobial susceptibility testing was performed on fresh colonies at the NDSU Veterinary Diagnostic Laboratory as described previously (30). The minimum inhibitory concentrations (MICs) of 41 antibiotics were determined by microdilution (Sensititre; Thermo Fisher Scientific, Nepean, ON, Canada) using commercially available panels [companion animal Gram-positive (COMPGP1F), equine (EQUIN1F), bovine (BOPO7F), Trek Diagnostic Systems, Cleveland, OH, USA] . The AST set-up and procedure were performed as suggested for these panels. The Sensititre AIM delivery system was used to inoculate to the 96-well AST plates, and the plates were incubated aerobically at 37°C for 24 h. After incubation, the plates were evaluated with a BIOMIC V3 system (Giles Scientific USA; Santa Barbara, CA, USA). Quality control was performed on the microdilution plates as recommended by the Clinical and Laboratory Standards Institute (CLSI) VET01S standards (32). Breakpoints for *Corynebacterium* isolates were determined using the criteria provided by CLSI document M45 (33). Breakpoints for all other isolates were determined using CLSI M100 (34).

### Statistical analysis

Bray-Curtis dissimilarities and permutational multivariate analysis of variance (PERMANOVA) using the adonis2 function in vegan in R were used to assess the vaginal and uterine microbial community structures in pregnant vs. open (non-pregnant) cattle. To identify fertility-associated taxonomic signatures, differentially abundant ASVs in the vaginal and uterine microbiota between pregnant and open cows and heifers were identified using the DESeq2 package (35). Only those ASVs found in at least 25% of all samples analyzed were included, and the samples were not randomly subsampled prior to in the DESeq2 analyses. Differentially abundant genera and phyla were also identified using MaAsLin2 v. 1.14.1 (36). Only those genera or phyla with an overall relative abundance of at least 0.1% were included in this analysis. For these analyses, a false discovery rate of less than 0.10 was used to identify differentially abundant taxa.

The number of ASVs (microbial richness) and microbial diversity metrics of the pregnant and open groups were compared using *t*-tests or the Mann-Whitney U test depending on the normality of the data using SAS (ver. 9.4, SAS Institute Inc., Cary, NC, USA). The Shapiro-Wilk test was used to determine whether a data set follows a normal distribution. Significance was considered at *P* < 0.05. Ecological network modeling was performed to compare the interaction network structure among all observed genera in uterine microbiota between open and pregnant cows using the generalized Lotka-Volterra models (gLVMs) by coupling biomass estimation and model inference with an expectation-maximization algorithm (BEEM) as described by Li et al. (37). The interaction network models (open and pregnant groups) created by BEEM statistic was visualized by using the graph package of R (38).

## RESULTS

### 16S rRNA gene sequencing overview

After processing and quality filtering, the average number of sequences per sample from the vaginal and uterine samples (*n* = 119) obtained from cows and heifers (vaginal swabs only, *n* = 60) were 58,928 ± 23,109 (SD) and 85,343 ± 23,422, respectively. From these sequences, a total of 29,165 ASVs representing 757 unique genera and 41 phyla were identified, although the large majority of these taxa were rare.

### Vaginal microbiota in heifers that became pregnant or remained open following AI

**Fig 1:**
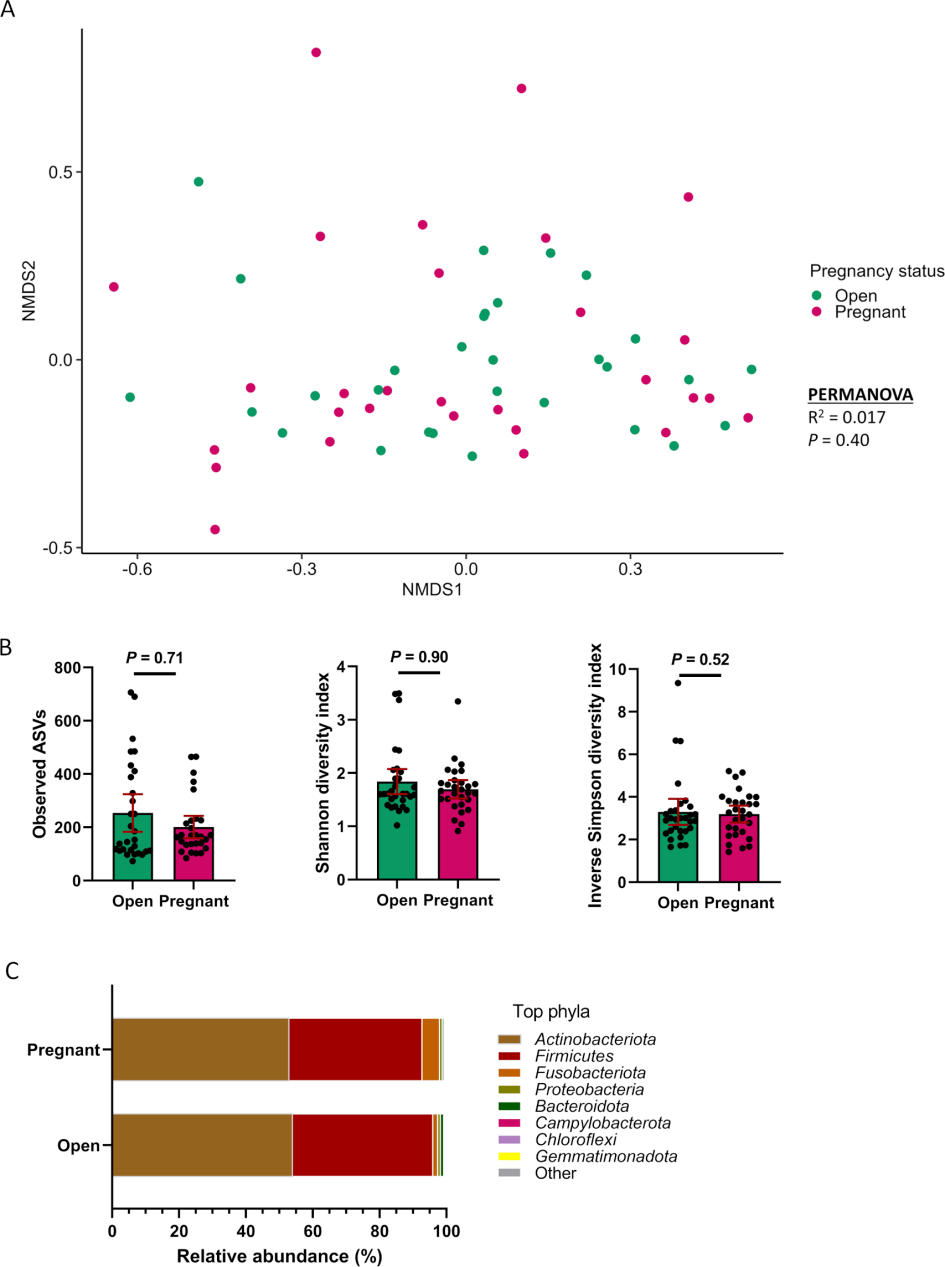
Beta and alpha diversities and composition (phylum level) of the vaginal microbiota of heifers that remained open (open, *n* = 33) or became pregnant (*n* = 26) following artificial insemination. (**A**) Non-metric multidimensional scaling (NMDS) plots of the Bray-Curtis dissimilarities, (**B**) number of observed amplicon sequence variants, and Shannon and inverse Simpson diversity indices; (**C**) percentage of relative abundance of the eight most relatively abundant phyla.

The community structure of the vaginal microbiota did not differ between the pregnant and open heifers at the time of AI (*R* ^2^ = 0.016; *P* = 0.42) (Fig. 1A). Pregnant heifers had similar microbial richness (observed ASVs) (Fig. 1B) and diversity (Shannon and inverse Simpson diversity indices; Fig. 1C and D) compared with open heifers (*P* > 0.05). Although no genera or phyla were differentially abundant between the two groups, DESeq2 identified 11 ASVs that were significantly enriched in the vaginas of heifers that later failed to become pregnant following AI compared with heifers that were successfully impregnated (*P* < 0.10) (Table 1). These ASVs were all within the phylum *Firmicutes*, although only four were classified at the genus level. The ASVs classified at the genus level were within the families *Lachnospiraceae* [*Dorea* [(ASV19), *Coprococcus* (ASV59), and UCG-010 (ASV30)], *Oscillospiraceae* [UCG-005 (ASV27 and 112)], *Oscillibacter* (ASV132), and *Butyricicoccaceae* [UCG-009 (ASV86)]. Overall, the *Trueperella*, *Helcococcus*, and *Peptoniphilus* genera were most relatively abundant in both groups of heifers (Table S1). Moreover, *Trueperella* spp. comprised greater than 50% of the 16S rRNA gene sequences in the vagina of both open and pregnant heifers.

**TABLE 1:** Differentially abundant ASVs in the vaginal microbiota of heifers between pregnant (*n* = 33) and open (*n* = 26) heifers^a^

ASV ID	baseMean	log_2_ fold change	lfcSE	adj *P* value	Phylum	Family	Genus
ASV17	43.7	2.5	0.87	0.0884	Firmicutes	[Eubacterium] coprostanoligenes group	NA
ASV19	60.6	2.8	0.91	0.0630	Firmicutes	Lachnospiraceae	Dorea
ASV27	42.2	4.6	1.49	0.0630	Firmicutes	Oscillospiraceae	UCG-005
ASV30	75.9	3.1	0.84	0.0371	Firmicutes	Lachnospiraceae	Lachnospiraceae UCG-010
ASV48	34.5	3.9	1.39	0.0891	Firmicutes	[Eubacterium] coprostanoligenes group	NA
ASV56	23.1	3.9	1.39	0.0884	Firmicutes	_Oscillospirales f_UCG-010	NA
ASV59	9.9	3.2	1.15	0.0884	Firmicutes	Lachnospiraceae	Coprococcus
ASV68	25.4	3.6	1.18	0.0630	Firmicutes	Butyricicoccaceae	UCG-009
ASV112	17.5	3.8	1.26	0.0630	Firmicutes	Oscillospiraceae	UCG-005
ASV132	17.0	3.6	1.18	0.0630	Firmicutes	Oscillospiraceae	Oscillibacter
ASV185	12.4	3.8	1.40	0.0982	Firmicutes	_Oscillospirales f_UCG-010	NA

^a^ Negative fold change values indicate that an ASV was more abundant in open heifers.

### Vaginal microbiota in cows that became pregnant or remained open following AI

**Fig 2:**
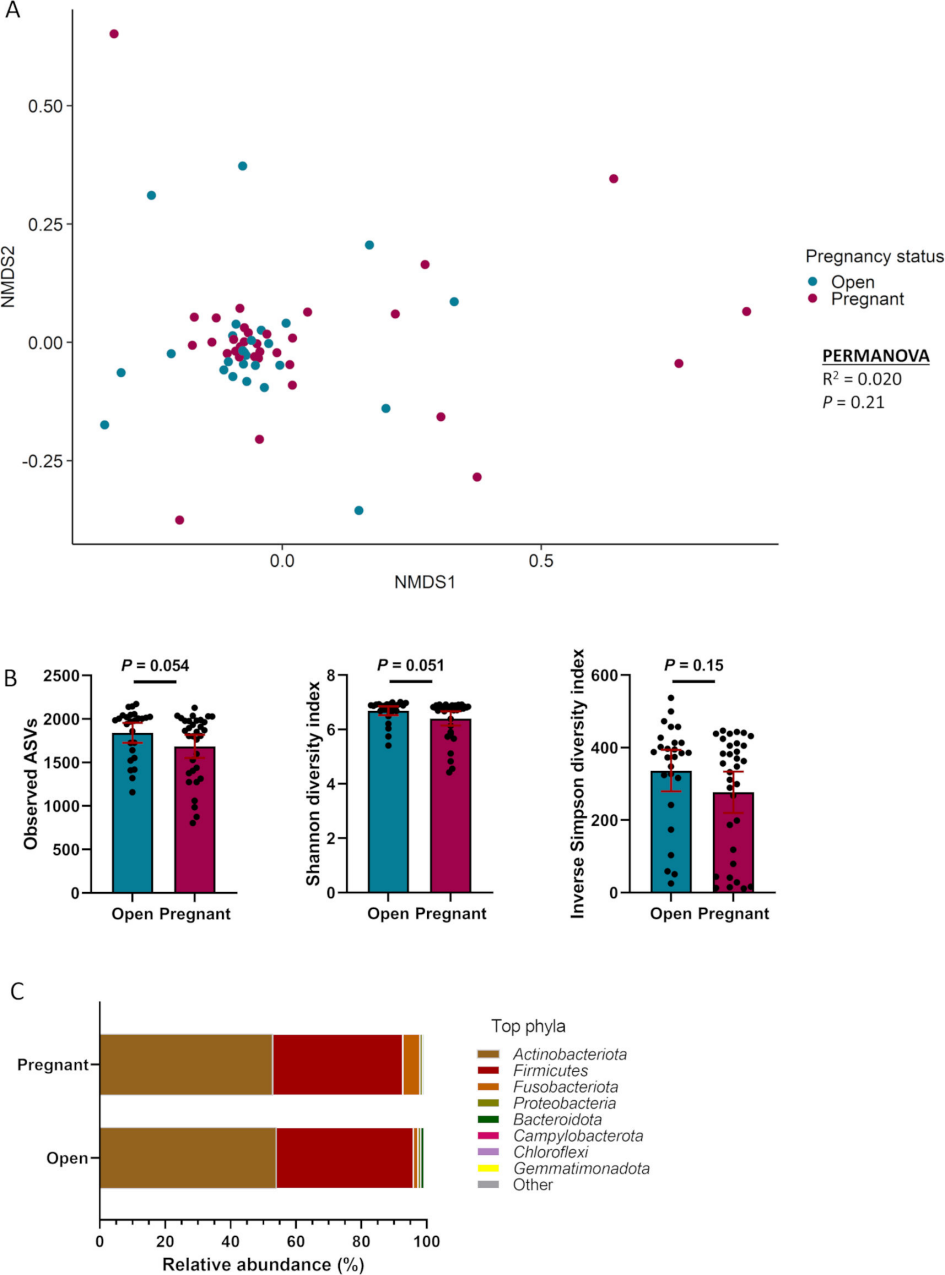
Beta and alpha diversities and composition (phylum level) of the vaginal microbiota of cows that remained open (open, *n* = 31) or became pregnant (*n* = 29) following artificial insemination. (**A**) Non-metric multidimensional scaling plots of the Bray-Curtis dissimilarities, (**B**) number of observed amplicon sequence variants, and Shannon and inverse Simpson diversity indices; (**C**) percentage of relative abundance of the eight most relatively abundant phyla.

As with the heifers, the vaginal microbiota community structure was similar for pregnant and open cows at the time of AI (*R* ^2^ = 0.020, *P* = 0.21) (Fig. 2A). Open cows tended to have greater microbial richness (*P* = 0.054) and Shannon diversity (*P* = 0.051) compared with cows that became pregnant (Fig. 2B). Overall, the vaginal bacterial community in cows was predominantly colonized by the phyla Actinobacteriota and Firmicutes which accounted for almost 90% of the total community composition (Fig. 2C). Their relative abundance did not differ between cows that became pregnant or remained open (*P* > 0.05). No ASVs, genera, or phyla were differentially abundant between the pregnant and open cows (*P* > 0.1). The uncultured genera *Oscillospiraceae* UCG-005 and *Rikenellaceae* RC9 gut group were the most relatively abundant genera in the vaginal microbiota of the cows (Table S2).

### Uterine microbiota in cows that became pregnant or remained open following AI

**Fig 3:**
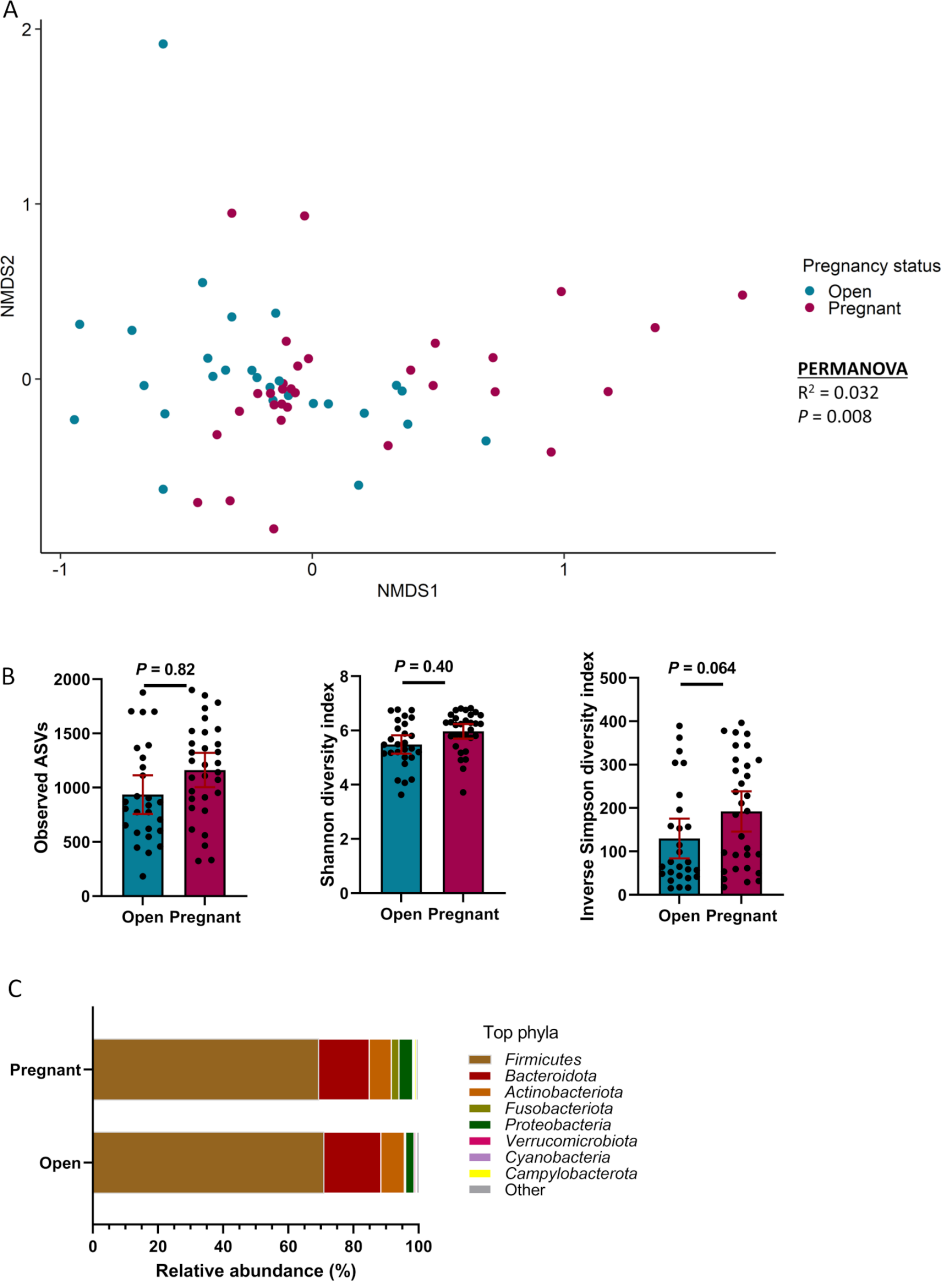
Beta and alpha diversities and composition (phylum level) of the uterine microbiota of cows that remained open (*n* = 31) or became pregnant (*n* = 29) following artificial insemination. (**A**) Non-metric multidimensional scaling plots of the Bray-Curtis dissimilarities, (**B**) number of observed amplicon sequence variants, and Shannon and inverse Simpson diversity indices; (**C**) percentage of relative abundance of the eight most relatively abundant phyla.

**Fig 4:**
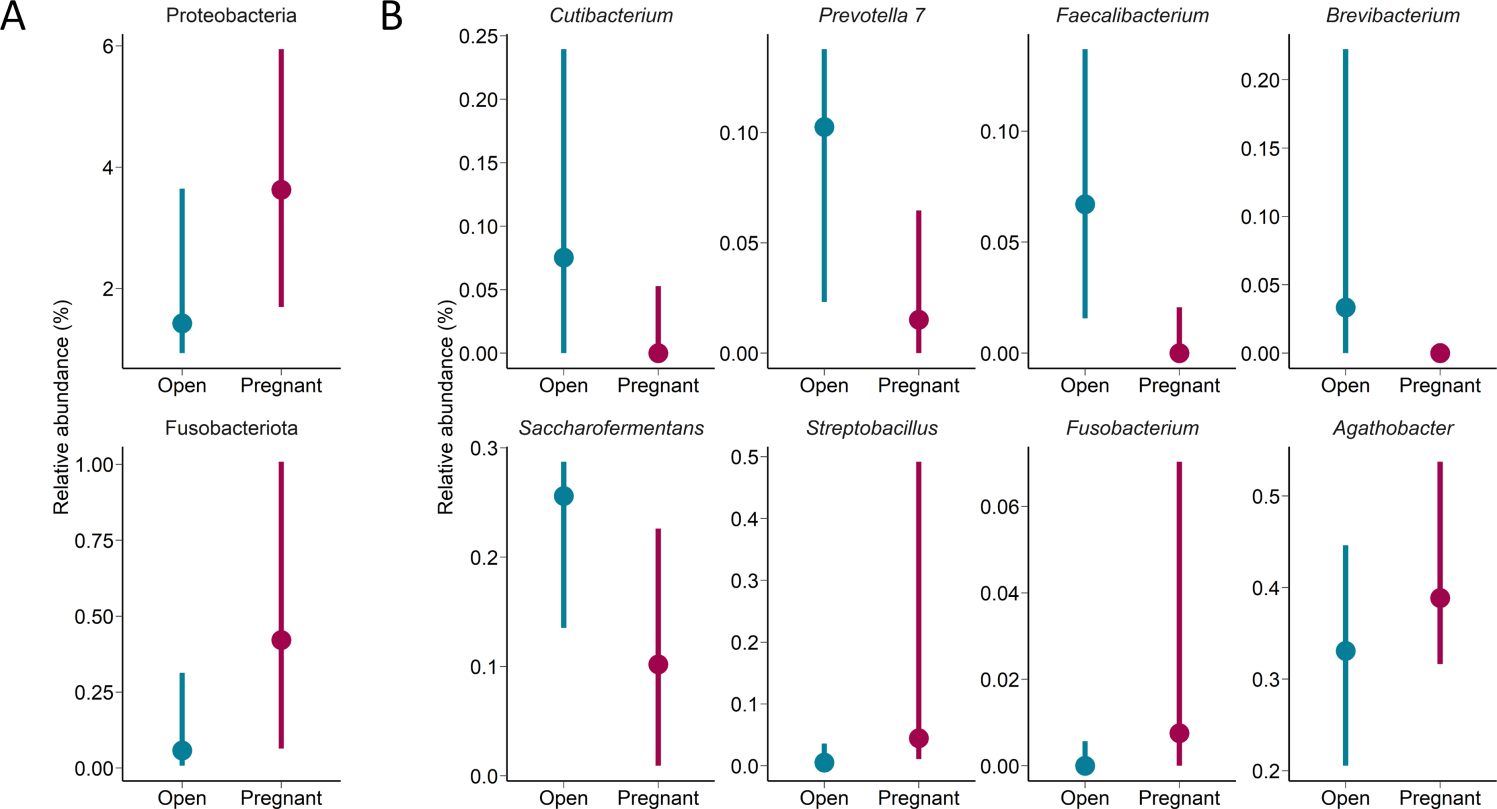
Differentially abundant (**A**) phyla and (**B**) genera in the uterine microbiota between cows that remained open or became pregnant following artificial insemination (*P* < 0.10). Displayed are the upper (75%) and lower (25%) quantiles and the median percent relative abundance for each taxon.

Cows that became pregnant following AI had a distinct uterine microbiota community structure (*R* ^2^ = 0.032, *P* = 0.008) (Fig. 3A) compared with those that remained open. However, the microbial richness (observed ASVs), Shannon diversity (*P* ≥ 0.40; Fig. 3B), and inverse Simpson’s diversity index (*P* = 0.064) did not differ in the pregnant group compared with the open group (Fig. 3B). Overall, the uterine bacterial community in cows was dominated by the phylum Firmicutes whose relative abundance accounted for almost 70% of the 16S rRNA gene sequences (Fig. 3C). The relative abundance of the phyla Fusobacteria and Proteobacteria was greater in the cows that became pregnant (Fig. 4A). The genera *Agathobacter*, *Fusobacterium*, and *Streptobacillus* were also more relatively abundant in the uterine microbiota of pregnant cows while *Brevibacterium*, *Cutibacterium*, *Faecalibacterium*, *Prevotella* 7, and *Saccharofermentans* were enriched in the cows that remained open (Fig. 4B). Twenty-eight differentially abundant ASVs were identified between the pregnant and open groups, with 11 of them more abundant in pregnant cows (Table 2). *Methanobrevibacter ruminantium* (archaea) and *Fusobacterium necrophorum* were among these 11 fertility-associated ASVs.

**TABLE 2:** Differentially abundant ASVs in the uterine microbiota of cows that became pregnant (*n* = 31) or remained open (*n* = 27)^a^ following artificial insemination

ASV ID	baseMean	log_2_ fold change	lfcSE	adj *P* value	Phylum	Family	Genus	Species
ASV644	16.7	5.64	1.33	0.0053	Firmicutes	*Monoglobaceae*	*Monoglobus*	NA
ASV1493	11.2	4.41	1.34	0.0456	Firmicutes	*[Eubacterium] coprostanoligenes group*	NA	NA
ASV592	27.9	4.24	1.36	0.0747	Firmicutes	*Oscillospiraceae*	*UCG-005*	NA
ASV957	30.8	4.11	1.15	0.0194	Firmicutes	*Ruminococcaceae*	*Faecalibacterium*	*prausnitzii*
ASV612	10.0	3.96	0.90	0.0036	Firmicutes	*Christensenellaceae*	Christensenellaceae R-7 group	NA
ASV866	12.1	3.55	1.20	0.0930	Actinobacteriota	NA	NA	NA
ASV385	21.2	3.47	1.12	0.0747	Bacteroidota	*Prevotellaceae*	*Prevotella_7*	NA
ASV682	19.7	3.42	1.19	0.0970	Firmicutes	*Lachnospiraceae*	*[Ruminococcus] gauvreauii group*	NA
ASV506	13.9	2.74	0.95	0.0970	Bacteroidota	*Rikenellaceae*	*Rikenellaceae RC9 gut group*	NA
ASV401	12.9	2.49	0.81	0.0753	Firmicutes	*Oscillospiraceae*	*UCG-002*	NA
ASV103	63.7	2.04	0.52	0.0083	Bacteroidota	*Prevotellaceae*	*Prevotellaceae UCG-004*	NA
ASV604	17.1	2.01	0.70	0.0970	Firmicutes	*UCG-010*	NA	NA
ASV321	22.4	1.95	0.53	0.0159	Firmicutes	*Oscillospiraceae*	*UCG-005*	NA
ASV225	43.3	1.85	0.49	0.0115	Firmicutes	*[Eubacterium] coprostanoligenes group*	NA	NA
ASV177	44.0	1.40	0.48	0.0970	Firmicutes	*[Eubacterium] coprostanoligenes group*	NA	NA
ASV57	121.7	1.34	0.46	0.0970	Firmicutes	*Oscillospiraceae*	*UCG-005*	NA
ASV77	157.9	1.07	0.35	0.0758	Firmicutes	*Lachnospiraceae*	*Shuttleworthia*	NA
ASV44	145.9	0.78	0.24	0.0418	Firmicutes	*[Eubacterium] coprostanoligenes group*	NA	NA
ASV288	39.6	0.91	0.30	0.0758	Firmicutes	*UCG-010*	NA	NA
ASV308	33.3	1.84	0.63	0.0970	Firmicutes	*Oscillospiraceae*	*UCG-005*	NA
ASV452	35.1	1.92	0.63	0.0758	Firmicutes	*Oscillospiraceae*	*NK4A214 group*	NA
ASV205	63.1	2.34	0.61	0.0109	Firmicutes	*f__[Eubacterium] coprostanoligenes group*	NA	NA
ASV2694	9.2	3.84	1.15	0.0418	Proteobacteria	NA	NA	NA
ASV330	16.7	3.86	1.08	0.0194	Euryarchaeota	*Methanobacteriaceae*	*Methanobrevibacter*	*ruminantium*
ASV16	167.8	4.56	1.14	0.0083	Fusobacteriota	*Leptotrichiaceae*	NA	NA
ASV1255	21.9	4.91	1.34	0.0159	Proteobacteria	NA	NA	NA
ASV12	28.0	5.36	1.33	0.0083	Fusobacteriota	*Fusobacteriaceae*	*Fusobacterium*	*necrophorum*
ASV446	22.4	7.19	1.30	0.0000	Proteobacteria	NA	NA	NA

^a^ Positive log_2_ fold change values indicate that an ASV was more abundant in the pregnant cows and vice versa.

### Interaction of the network structure of the uterine microbiota between open and pregnant cows

**Fig 5:**
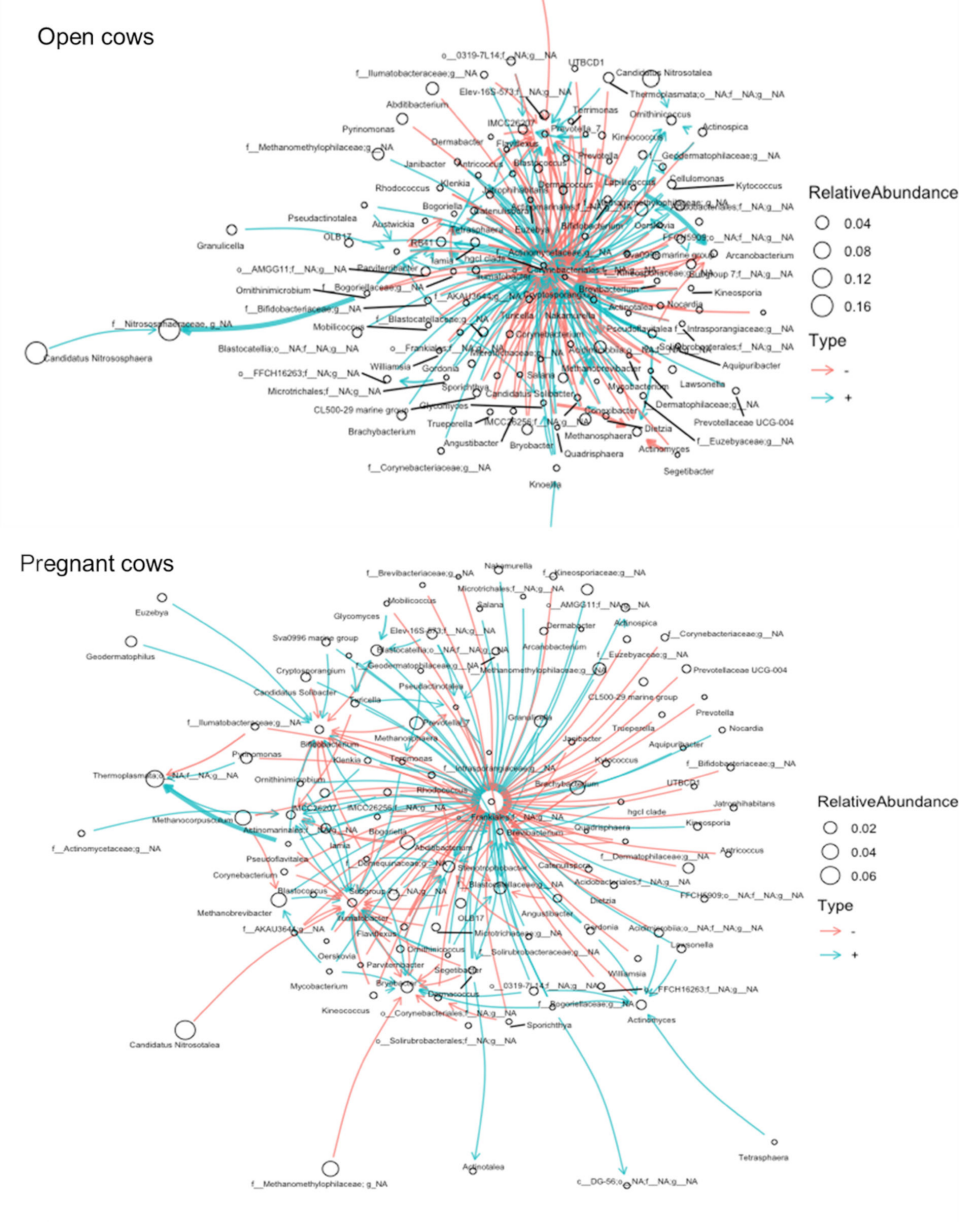
Ecological network of observed bacterial genera in the uterine microbiota of cows that remained open (*n* = 31) or became pregnant (*n* = 29) following artificial insemination.

After observing the community structure and compositional differences in the uterine microbiota between the two groups of cows, we next used ecological network modeling to analyze directed interactions among all observed genera. As shown in the network plots (Fig. 5), cows that failed to become pregnant via AI had a noticeably distinct interaction network structure as compared with cows that became pregnant. Compared with the network structure observed in the open group, the complexity of the interaction network of the uterine microbiota from pregnant cows was reduced with a lower number of total genera that remained in the model. Despite having a less intense interaction network structure, the total number of hubs connecting the interactions between genera was greater than the total number of hubs observed in the network structure of the open group (eight vs. three hubs). Overall, an equal proportion of positive and negative infractions between genera was detected in both network models.

### Comparing the vaginal and uterine microbiota in cows

**Fig 6:**
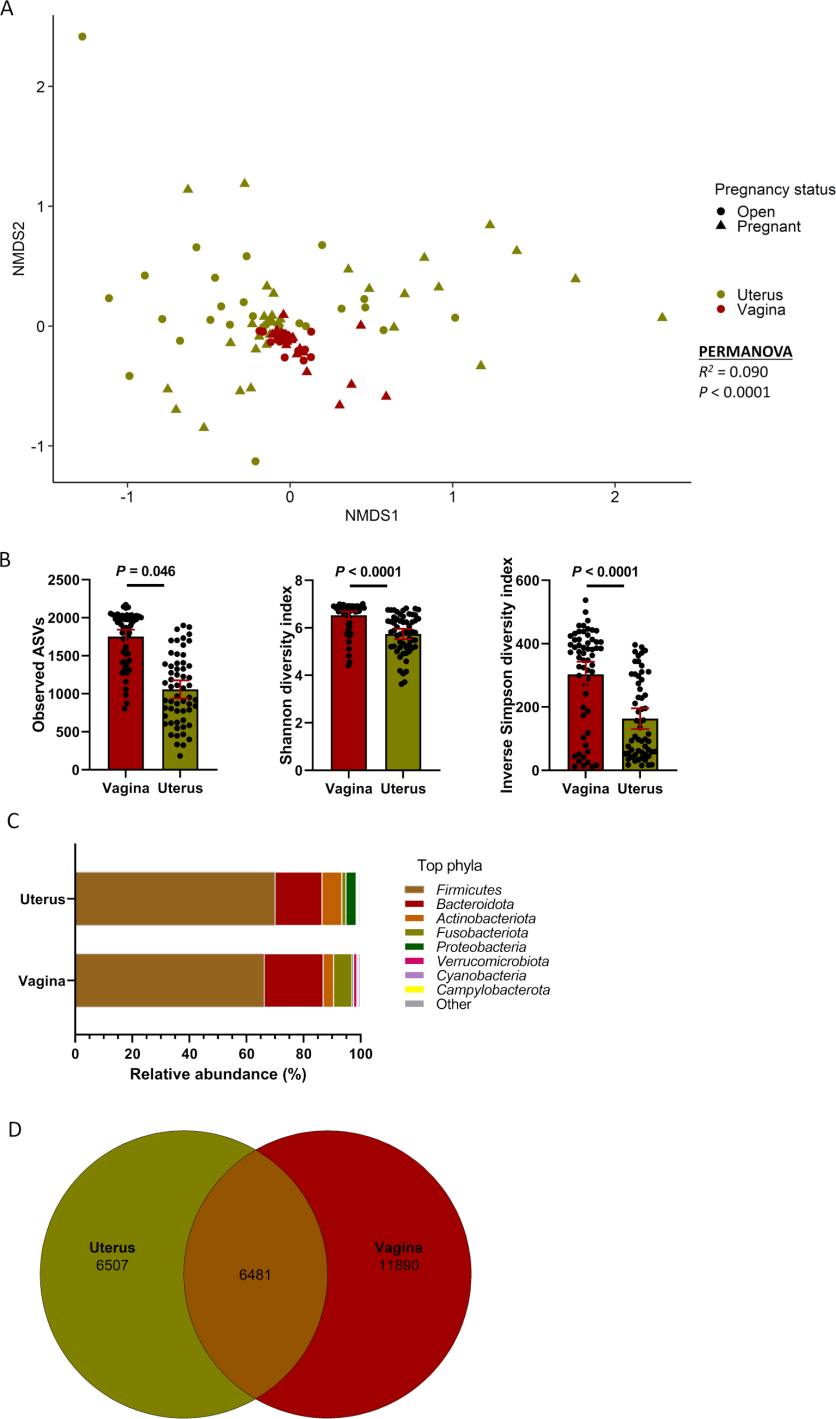
Beta and alpha diversities and composition (phylum level) of the vaginal and uterine microbiota from cows (*n* = 60) that become pregnant or remained open following artificial insemination. (**A**) Non-metric multidimensional scaling plots of the Bray-Curtis dissimilarities, (**B**) number of observed amplicon sequence variants, and Shannon and inverse Simpson diversity indices; (**C**) percentage of relative abundance of the eight most relatively abundant phyla; (**D**) Venn diagram displaying the number of shared and unique ASVs between uterine and vaginal microbiota.

**Fig 7:**
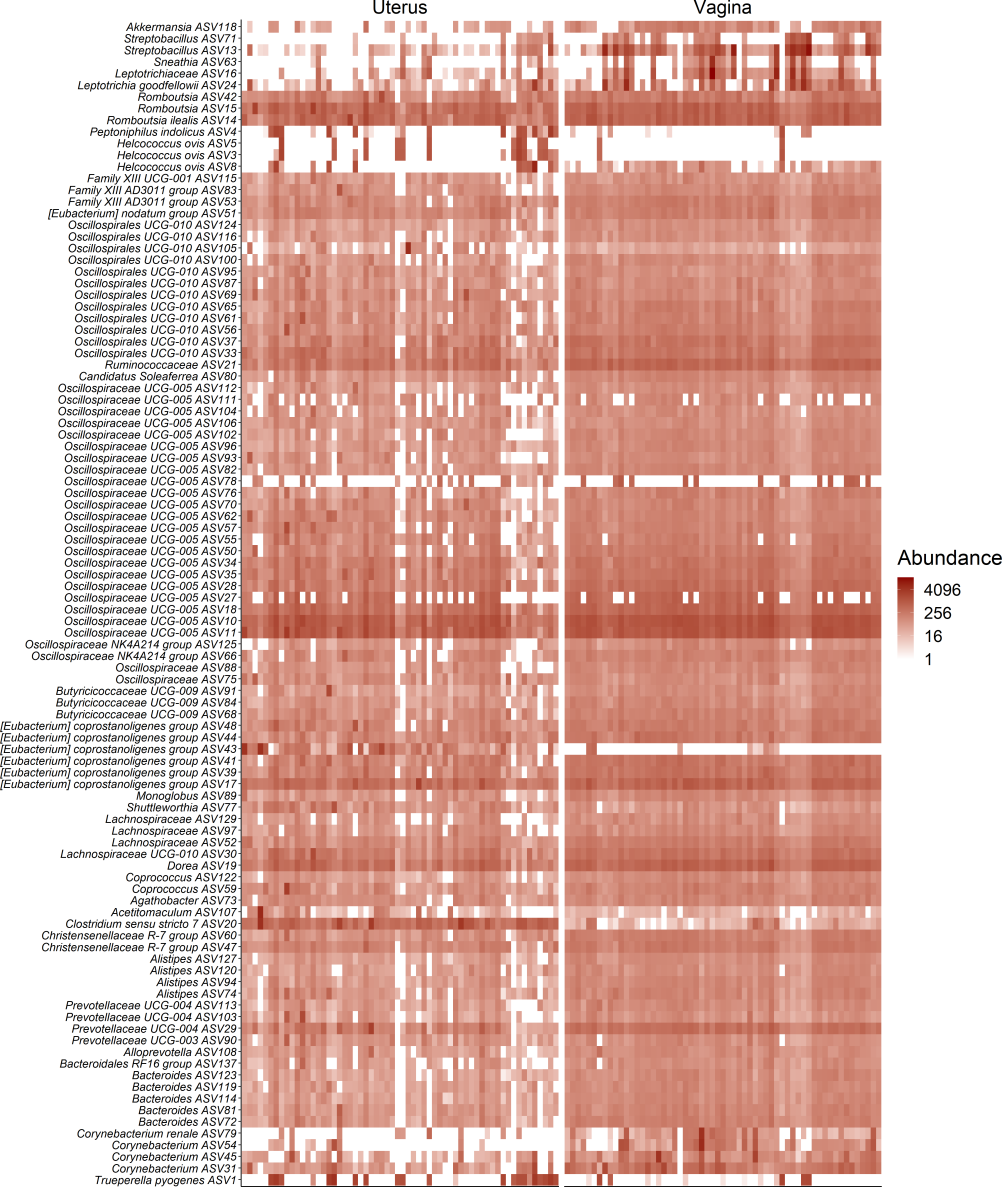
Heat map showing the 100 most abundant ASVs (log4) overall in the uterine and vaginal microbiota of cows (*n* = 60) that become pregnant or remained open following artificial insemination.

Given that vaginal and uterine swabs were collected simultaneously from the same animal, we compared the microbial composition and diversity between the cow vaginal and uterine microbiota. Overall, the microbial community in the uterus was distinct from that of the vagina in terms of structure (*R* ^2^ = 0.09; *P* < 0.0001) (Fig. 6A), richness, and diversity (*P* ≤ 0.04) (Fig. 6B). As expected, the diversity and richness of the vaginal microbiota were significantly greater than the uterine microbiota. Both reproductive organs were colonized by the same dominant bacteria phyla but the relative abundance of some of them (Bacteroidota, Actinobacteriota, and Fusobacteriota) varied between the two sites (Fig. 6C). Despite these differences, a total of 6,481 ASVs (26% of overall ASVs) were shared between the vaginal and uterine microbiota (Fig. 5D). As shown in the heatmap (Fig. 7), the vast majority of the 100 most abundant ASVs were present in both the vaginal and uterine microbiota with a similar frequency and abundance. Only a few ASVs, such as ASV8 (*Helcococcus ovis*), ASV79 (*Corynebacterium renale*), and ASV54 (*Corynebacterium*), were almost exclusively present in the uterus. An *Eubacterium coprostanoligenes* group ASV (ASV43) was the only taxon exclusively found in vaginal samples. Overall, it is clear that a significant number of bacterial taxa are present in both the vagina and uterus of cattle.

### Comparing the vaginal microbiota between heifers and cows

**Fig 8:**
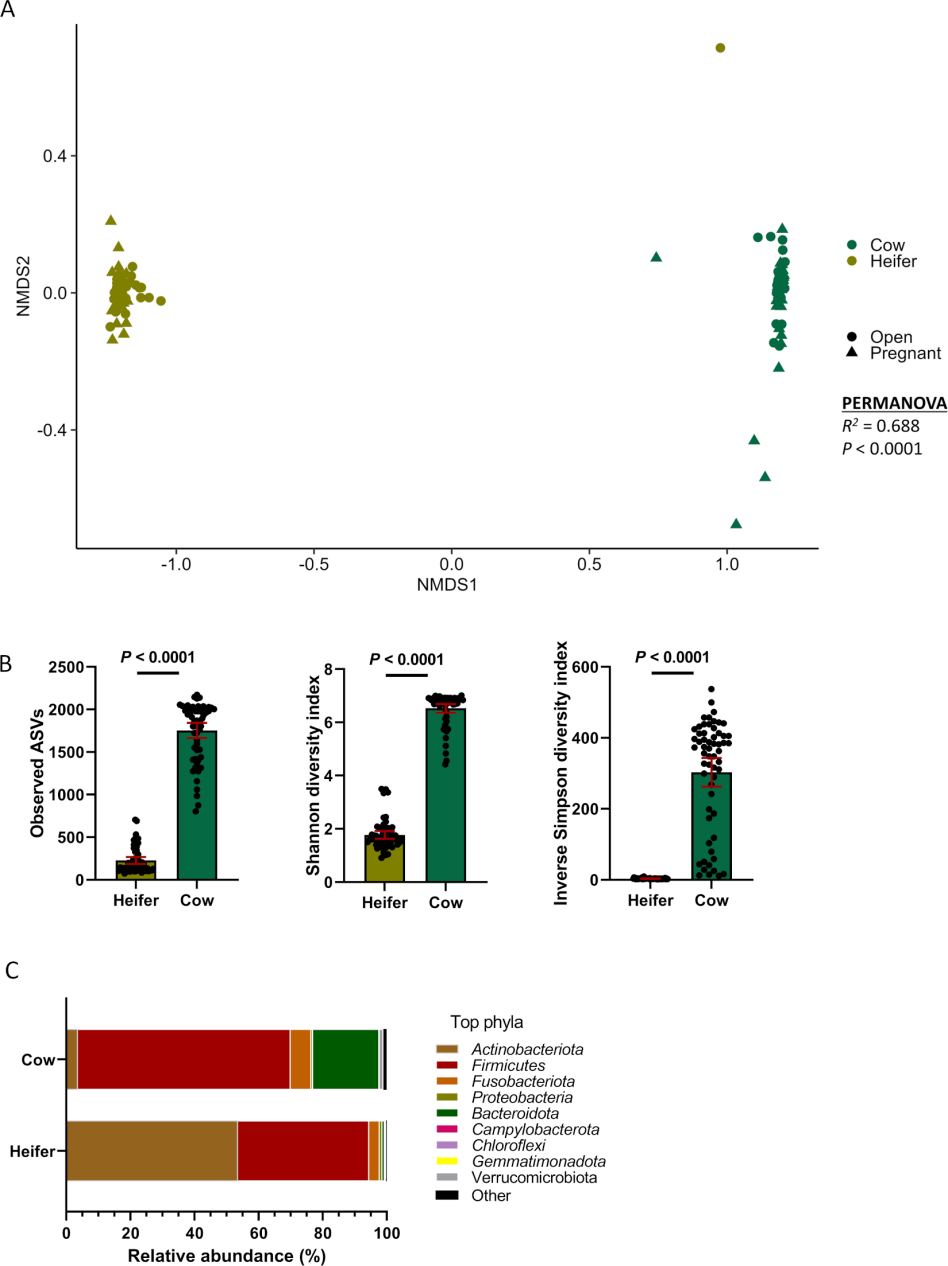
Beta and alpha diversities and composition (phylum level) of the vaginal microbiota between heifers (*n* = 59) and cows (*n* = 60). (**A**) Non-metric multidimensional scaling plots of the Bray-Curtis dissimilarities, (**B**) number of observed amplicon sequence variants, and Shannon and inverse Simpson’s diversity index. (**C**) Percentage of relative abundance of the eight most relatively abundant phyla.

There was a significant and very large difference in the community structure of the vaginal microbiota between cows and heifers (*R* ^2^ = 0.688; *P* < 0.001) (Fig. 8A). Vaginal microbial species richness and diversity were much greater in cows than heifers (Fig. 8B). Such differences were also reflected in the microbial composition, particularly the relative abundance of most relatively abundant bacterial phyla. The vaginas of cows were predominantly colonized by species within the phyla Firmicutes and Bacteroidota, whereas the vaginas of heifers were dominated equally by Firmicutes and Actinobacteriota.

### Isolation and identification of vaginal and uterine bacterial isolates using aerobic and anaerobic culturing

#### Vaginal bacterial isolates

**Fig 9:**
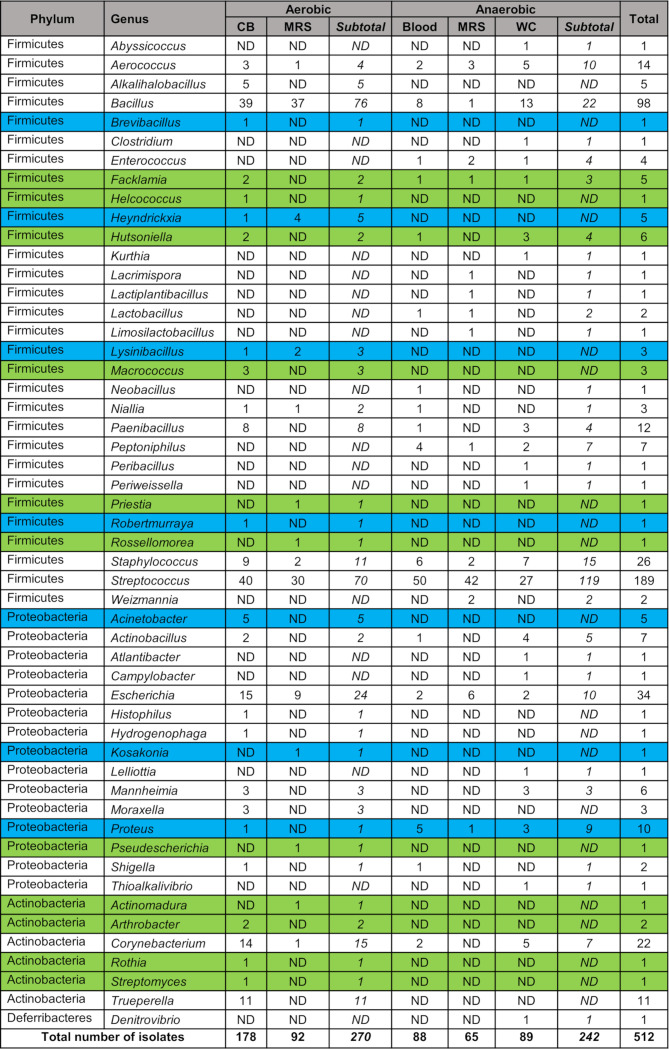
Bacterial isolates from aerobic and anaerobic culturing of vaginal swab samples collected from yearling heifers and cows of various ages. Blue-highlighted genera were only present in heifers, and green-highlighted genera were only found in cows.

The vaginal swabs of 84 animals were plated aerobically and anaerobically which resulted in the recovery of 512 bacterial isolates, representing 52 genera within the phyla Firmicutes (58%), Proteobacteria (28%), Actinobacteria (12%), and Deferribacterota (2%) (Fig. 9). *Streptococcus* (37%), *Bacillus* (19%), *Escherichia* (7%), *Staphylococcus* (5%), and *Corynebacterium* (4%) were the most frequently isolated genera. From the aerobic culturing of 64 vaginal swabs plated on MRS (semi-selective for lactic acid bacteria) and CB (non-selective) agar plates, 270 bacterial isolates (MRS = 92; CB = 178 isolates) were recovered (Fig. 9). These isolates were mainly from three phyla, Firmicutes (73%), Proteobacteria (16%), and Actinobacteria (11%), and 34 different genera with *Bacillus* (28%), *Streptococcus* (26%), *Escherichia* (9%), *Corynebacterium* (6%), *Trueperella* (4%), and *Staphylococcus* (4%) being the most frequently isolated.

Thirty-four vaginal swabs were plated and cultured anaerobically on blood, MRS, and WC agar plates resulting in the recovery of 242 bacterial isolates (Fig. 9). The 88 isolates recovered from blood plates were taxonomically assigned to 17 genera from three phyla: Firmicutes (88%), Proteobacteria (10%), and Actinobacteria (2%). The isolates recovered from MRS plates (*n* = 65) were from 14 different genera within the Firmicutes (89%) and Proteobacteria (11%) phyla. The WC plates yielded slightly more diverse bacterial species (*n* = 89) comprised of 24 different genera from four different phyla: Firmicutes (75%), Proteobacteria (18%), Actinobacteria (6%), and Deferribacterota (1%). Overall, 32 different genera within four phyla, Firmicutes (83%), Proteobacteria (13%), Actinobacteria (3%), and Denitrovibrio (1%), were recovered from anaerobic culturing. The five most prevalent genera recovered were *Streptococcus* (49%), *Bacillus* (9%), *Staphylococcus* (6%), *Aerococcus* (4%), and *Escherichia* (4%).

#### Uterine bacterial isolates

Overall, 221 bacterial isolates were recovered from the uterine swabs (*n* = 44) that were cultured aerobically and anaerobically (Table 3). These isolates were from 29 different genera within the Firmicutes (52%), Proteobacteria (34%), and Actinobacteria (14%) phyla. *Streptococcus* (48%), *Bacillus* (15%), *Escherichia* (6%), *Corynebacterium* (5%), and *Facklamia* (3%) were the predominant genera. Twenty-four uterine swabs were cultured aerobically on CB and MRS agar plates (Table 3) yielding 93 bacterial isolates representing 21 genera. The five most prevalent genera were *Streptococcus* (30%), *Bacillus* (26%), *Escherichia* (11%), *Staphylococcus* (5%), and *Moraxella* (4%). The remaining 128 isolates were from 34 uterine swabs after anerobic culturing on blood, MRS, and WC agar plates (Table 3). These isolates were from 17 bacterial genera within the Firmicutes (81%), Proteobacteria (10%), and Actinobacteria (9%) phyla. The most frequently isolated genera were *Streptococcus* (62%), *Bacillus* (8%), *Corynebacterium* (7%), *Facklamia* (4%), *Limosilactobacillus* (3%), *Actinobacillus* (3%), and *Escherichia* (3%).

**TABLE 3:** Bacterial isolates from aerobic and anaerobic culturing of uterine swab samples collected from cows of various ages

Phylum	Genus	Aerobic			Anaerobic				Total
		CB	MRS	Subtotal	Blood	MRS	WC	Subtotal	
Firmicutes	*Aerococcus*	1	ND	*1*	ND	ND	1	*1*	2
Firmicutes	*Bacillus*	12	12	*24*	7	ND	3	*10*	34
Firmicutes	*Caldibacillus*	ND	ND	*ND*	ND	1	ND	*1*	1
Firmicutes	*Enterococcus*	ND	ND	*ND*	1	2	ND	*3*	3
Firmicutes	*Erysipelothrix*	ND	ND	*ND*	1	ND	ND	*1*	1
Firmicutes	*Facklamia*	2	ND	*2*	3	2	ND	*5*	7
Firmicutes	*Limosilactobacillus*	ND	ND	*ND*	1	3	ND	*4*	4
Firmicutes	*Lacticaseibacillus*	1	ND	*1*	ND	ND	ND	*ND*	1
Firmicutes	*Macrococcus*	ND	1	*1*	ND	ND	ND	*ND*	1
Firmicutes	*Niallia*	1	ND	*1*	ND	ND	ND	*ND*	1
Firmicutes	*Paenibacillus*	1	ND	*1*	ND	ND	ND	*ND*	1
Firmicutes	*Priestia*	1	1	*2*	ND	ND	ND	*ND*	2
Firmicutes	*Solibacillus*	1	ND	*1*	ND	ND	ND	*ND*	1
Firmicutes	*Staphylococcus*	5	ND	*5*	ND	ND	ND	*ND*	5
Firmicutes	*Streptococcus*	17	11	*28*	30	27	22	*79*	107
Proteobacteria	*Actinobacillus*	1	ND	*1*	ND	ND	4	*4*	5
Proteobacteria	*Escherichia*	6	4	*10*	ND	2	2	*4*	14
Proteobacteria	*Gallibacterium*	2	ND	*2*	ND	1	1	*2*	4
Proteobacteria	*Histophilus*	1	ND	*1*	ND	ND	ND	*ND*	1
Proteobacteria	*Mannheimia*	ND	ND	*ND*	ND	ND	1	*1*	1
Proteobacteria	*Moraxella*	4	ND	*4*	ND	ND	ND	*ND*	4
Proteobacteria	*Pelistega*	1	ND	*1*	ND	ND	ND	*ND*	1
Proteobacteria	*Pseudomonas*	2	ND	*2*	ND	ND	ND	*ND*	2
Proteobacteria	*Neisseria*	ND	ND	*ND*	ND	ND	1	*1*	1
Proteobacteria	*Shigella*	ND	1	*1*	ND	ND	1	*1*	2
Actinobacteria	*Arcanobacterium*	ND	ND	*ND*	ND	1	ND	*1*	1
Actinobacteria	*Corynebacterium*	3	ND	*3*	3	ND	6	*9*	12
Actinobacteria	*Kribbella*	ND	ND	*ND*	1	ND	ND	*1*	1
Actinobacteria	*Trueperella*	1	ND	*1*	ND	ND	ND	*ND*	1
Total number of isolates		63	30	*93*	47	39	42	*128*	221

### Antimicrobial susceptibility testing of selected vaginal and uterine isolates

**Fig 10:**
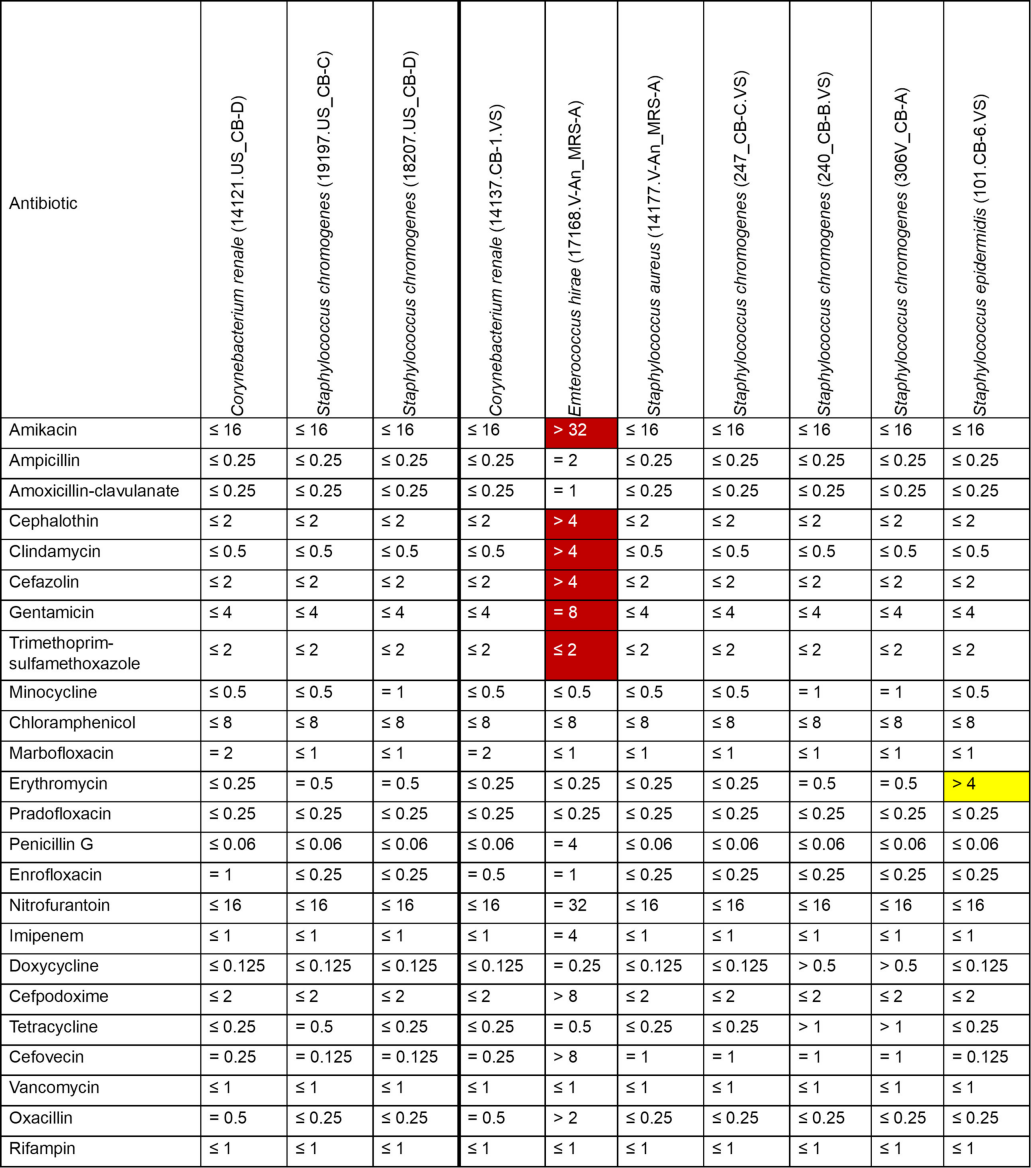
MICs of antibiotics against Gram-positive bacterial isolates (*n* = 10) isolated from the uterus (*n* = 3) and vagina (*n* = 7) of beef cows and heifers. MIC values (µg/mL) presented in the table were obtained from the Sensititre Gram Positive Companion panel (COMPGP1F). Green, no break points (isolate); blue, no/few break points (antibiotic); red, resistant; dark-red/white text, intrinsically resistant; yellow, highest test concentrations were lower than breakpoints; range, intermediate.

**Fig 11:**
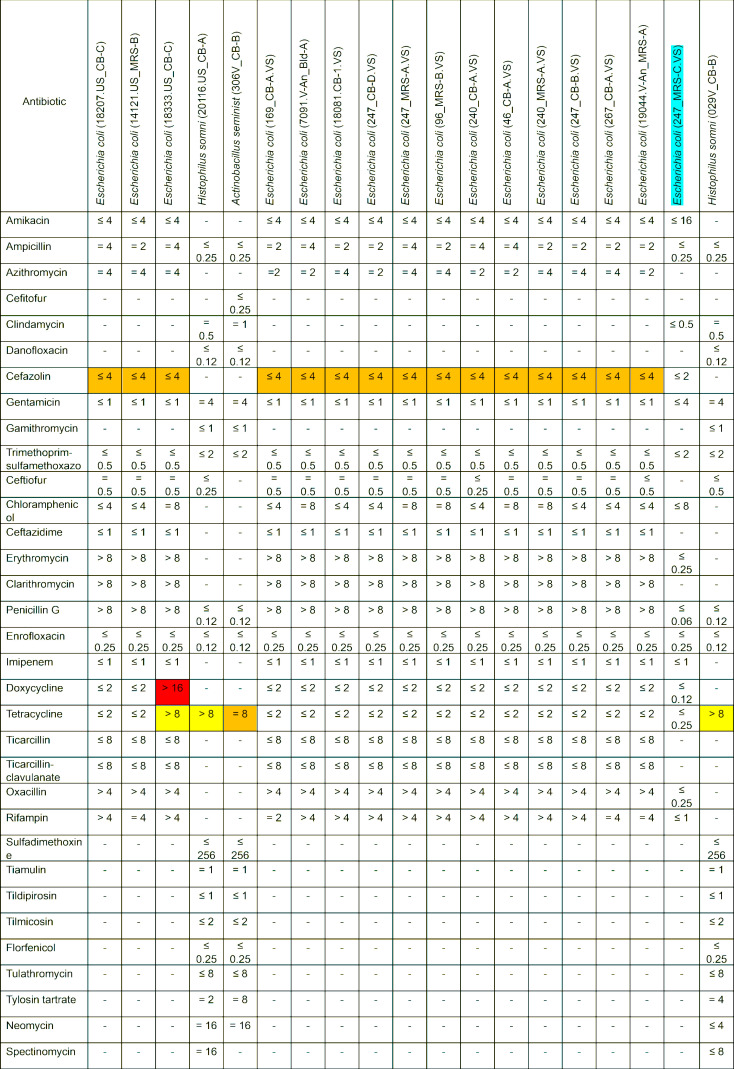
MICs of antibiotics against Gram-negative bacterial isolates (*n* = 19) isolated from the uterus (*n* = 4) and vagina (*n* = 15) of beef cows and heifers. MIC values (µg/mL) presented in the table were obtained from the Sensititre Gram Negative Companion panel (COMPGN1F). Green, no break points (isolate); blue, no/few break points (antibiotic); red, resistant; dark red/white text, intrinsically resistant; yellow, highest test concentrations were lower than breakpoints; orange intermediate.

The MICs of 41 different antibiotics from three different antibiotic panels were tested for 29 bacterial isolates (10 Gram-positive and 19 Gram-negative) from six different genera (Fig. 10 and 11). Based on the CLSI breakpoints, most of the Gram-positive isolates were susceptible to all antibiotics tested, with *Staphylococcus epidermidis* (101.CB-6.VS) potentially showing resistance to erythromycin (Fig. 10). *Enterococcus hirae* 17168.V-An_MRS-A was resistant to amikacin, cephalothin, cefazolin, trimethoprim-sulfamethoxazole, clindamycin, and gentamicin. Among Gram-negative isolates tested, most *Escherichia coli* isolates exhibited intermediate resistance to cefazolin (Fig. 11), and one of them (18333.US_CB-C) was also resistant to doxycycline. The remaining Gram-negative isolates were susceptible to most of the antibiotics tested. Of note, several isolates such as *S. epidermidis* (101.CB-6.VS), *E. coli* (18333.US_CB-C), and *Histophilus somni* (20116.US_CB-A and 029V_CB-B) were not inhibited at the maximum concentration for any of the antibiotics included in the panel, making it challenging to identify resistance breakpoints. There are no available breakpoints for *Actinobacillus seminist* (306V_CB-B) for any of the 20 antibiotics tested. Five antibiotics that were tested on the Gram-positive panel (amikacin, marbofloxacin, pradofloxacin, enrofloxacin, and imipenem) and 17 antibiotics from the Gram-negative panel (ceftiofur, clindamycin, danofloxacin, gamithromycin, erythromycin, clarithromycin, penicillin G, enrofloxacin, sulfadimethoxine, tiamulin, tildipirosin, tilmicosin, florfenicol, tulathromycin, tylosin tartrate, neomycin, and spectinomycin) had few to no breakpoint values documented for those isolates that we tested. Therefore, resistance to these antibiotics could not be interpreted.

## DISCUSSION

Overall, 16S rRNA gene sequencing revealed that the structure of the vaginal microbiota at the time of AI was not different between cows and heifers that became pregnant or remained open. However, the vaginal microbiota in pregnant cows tended to have lower richness and alpha diversity at the time of AI as compared with open cows. A vaginal microbiota with a reduced richness and diversity have been considered “normal and healthy” and positively associated with reproductive health, pregnancy outcome, and lower preterm birth risk in women (39 – 42). Thus, the lower microbial richness and diversity observed in the cow vaginal microbiota at the time of AI could be associated with the pregnancy rate. However, no differences were observed for any of the alpha diversity metrics of the vaginal microbiota in virgin heifers that had not previously been bred. This might be partially because the virgin heifers harbor a vaginal community with a significantly lower microbial richness and diversity as compared with non-virgin pregnant heifers (23) and cows with multiple parities and breeding histories (Fig. 7B). Using a larger number of virgin yearling heifers could have improved statistical power to detect differences in microbial richness and diversity between pregnant and open heifer groups. Thus, the association between the reduced microbial richness and diversity in yearling heifers and pregnancy rate warrants future study.

Moderate differences in the microbial composition at the ASV level was observed in the vagina between pregnant and open group heifers. The 11 ASVs that were inversely associated with AI pregnancy rates in heifers were from only three families: *Lachnospiraceae*, *Oscillospiraceae*, and *Butyricicoccaceae* (Table 3). In pregnant women allergic to antibiotics, the abundance of vaginal *Lachnospiraceae* was reduced as compared with that in non-allergic pregnant women (43). Both *Lachnospiraceae* and *Oscillospiraceae* were also found to be enriched in the vaginal microbiota of women with adenomyosis (44), a condition where the endometrial tissue that forms the lining of the uterus grows into the muscle of the uterine wall and enlarges the uterus (45). Together with our results, this suggests that a bovine vaginal microbiota with an increased proportion of *Lachnospiraceae* and *Oscillospiraceae* at the time of breeding could be associated with a reduced rate of conception. The *Butyricicoccaceae* family encompasses butyric acid-producing species in the gut (46), and butyric acid produced by bacteria has been shown to regulate animal reproduction by directly regulating progesterone and 17β-estradiol secretion (47). The elevated relative abundance of vaginal *Butyricicoccaceae UCG-009* in open heifers indicates that butyric acid-producing bacterial species in the reproductive tract may have a role in influencing bovine reproduction. Taken together, our results highlight that the abundance of certain bacterial species in the vaginal tract of female cattle before or at the time of breeding may influence pregnancy outcomes.

As an acceptable uterine swabbing technique for virgin yearling heifers was not available at the time of sampling, our uterine data were limited to cows. Compared with those in the vaginal microbiota, greater differences were observed in the uterine microbiota between cows that became pregnant and those that remained open. These differences include a distinct community structure and interaction network structure and several differentially abundant taxa between the two groups. Cows that became pregnant harbored noticeably different interaction networks among the observed genera compared with the open cows. Although it is challenging to make inferences on the biological meaning of ecological network modeling, active interactions with balanced positive (cooperation) and negative (competition) interconnectivity between different microbial species are important for maintaining the stability and functional features of the gut (48 – 50) and respiratory microbiota (38). Distinctive microbial relative co-abundance networks were observed in the vaginal microbiota between penicillin allergic and non-allergic women (43). Accordingly, our network results suggest that directionality of the interactions and cooperative and competitive interconnectivity between different uterine microbial species may have implications in female fertility and pregnancy.

A *F. necrophorum* ASV and the *Fusobacterium* genus were relatively more abundant in the uterus of cows that became pregnant which is surprising as this species is typically considered an opportunistic pathogen that is associated with several bovine diseases including liver abscesses and foot root, both of which have a negative effect on cattle performance and profitability in the beef cattle industry (51, 52). *F. necrophorum* has also been associated with abortion in cattle (53, 54). However, new evidence indicates that *F. necrophorum* in the bovine urogenital tract may not always be harmful but instead may be a commensal species with a positive role in reproductive health and fertility. For example, *F. necrophorum* was isolated in the seminal microbiota of healthy beef bulls (30). Recently, we identified *Fusobacterium* as one of the relatively abundant genera (0.6% relative abundance) in the uterus of healthy beef heifers (24) and in the semen (26%) of healthy bulls (30). In the present study, the uterus of pregnant cows harbored 125-fold greater relative abundance of *Fusobacterium* as compared with open cows at the time of AI (0.63% vs. 0.005%). According to a recent comparative genomic analysis of different *F. necrophorum* strains (55) and site-specific genome adaptations reported in *Lactobacillus* spp. (56), it is likely that *F. necrophorum* species that colonize the reproductive tract do not contain the virulence genes found in the pathogenic strains implicated in the liver-abscesses, foot rot, or abortion. To better understand the role of *F. necrophorum* in cattle reproductive health and fertility, a genomic comparison between the *F. necrophorum* isolates isolated from healthy cow uterus and from clinical infections should be the focus of future studies.

ASVs assigned to *Prevotella 7* (ASV385) and *Prevotellaceae* UCG-004 (ASV103) were significantly higher in open cow uterine samples, suggesting that certain *Prevotellaceae* spp. may negatively affect cattle fertility. ASV957, which was identified as *Faecalibacterium prausnitzii*, was more abundant in the uterus of open cows. Members of this species consume acetate and produce butyrate and other bioactive anti-inflammatory modulators (57). A higher proportion of *F. prausnitzii* in the uterus of open cows and the enrichment of vaginal *Butyricicoccaceae* UCG-009 in open heifers suggest that butyrate-producing bacteria in the urogenital tract of female cattle may have adverse effects on the reproductive health and pregnancy outcome. Open cows also harbored a greater relative abundance of ASVs identified as the *Rikenellaceae* RC9 gut group (ASV506) and *Christensenellaceae* R-7 group (ASV6112) in their uterus. Although both of these genera are part of the normal bovine gut microbiota (24), the *Rikenellaceae* RC9 gut group also colonizes the bovine uterus (18); however, there are no reports regarding their association with reproductive infections or fertility in cattle.

Overall, the vaginal microbial community structure, species richness, diversity, and composition were drastically different between heifers and cows. Cows harbored a significantly richer and more diverse vaginal microbiota dominated by species within the phylum Firmicutes as compared with heifers (Fig. 8). One acknowledged limitation with comparing the vaginal microbiota between heifers and cows here is samples from heifers were collected at the time of CIDR removal which was 2 days before AI and cows were sampled at the time of AI. Changes in progesterone and estrogen concentrations after CIDR removal could have a significant impact on the vaginal microbiota composition as reproductive hormones are known to influence the vaginal microbiome in cattle (20) and humans (58). However, the large differences observed in between the heifers and cows in terms of microbial community structure (PERMANOVA: *R* ^2^ = 0.69, *P* < 0.0001) and microbial richness and diversity highlight that other factors beyond reproductive hormones associated with the synchronization protocol could also be responsible. In a recent study, we characterized the vaginal microbiota associated with 30-h-old calves born from dams originating from the same farm as the cows used in the present study (59). These newborn calves harbored a vaginal microbiota with a lower richness (observed ASVs < 350) and diversity (Shannon diversity index < 3) compared with the vaginal microbiota of heifers and cows in the present study. These results indicate that cattle age, reproductive developmental stage, sexual activity, and number of births have a significant influence on the vaginal microbiota (60, 61). Apart from these factors, the housing environment and feed could be contributing to the different vaginal microbiota observed between heifers and cows as these two cohorts were housed in two different environments; cows were on the pasture while heifers were housed in an enclosed animal facility.

As expected, the structure, richness, and diversity of the vaginal and uterine microbiota in cows were significantly different from each other with the uterus having a less rich and diverse microbiota compared with the vagina. This is likely due to the physiological, immunological, microbiological, and anatomical differences in the vagina and uterus (62 – 64). Despite these differences, the vast majority of the 100 most abundant ASVs were present in both sites. Such similarities between the vaginal and uterine microbiota suggest that bacterial species at the mucosal surface of the vagina may ascend into the uterus despite differences in oxygen availability and other physiochemical properties between the two anatomical sites. Thus, future studies aimed at developing probiotics to enhance fertility and delivered *in utero* should consider those beneficial bacterial species present in the vaginal tract as well.

Considering that 89% and 57% of the differentially abundant ASVs in the uterine samples were not classified at the genus and species levels, respectively, and that a similar proportion of vaginal bacteria were not classified at the genus level in both heifers and cows, we also used comprehensive culturing to profile the vaginal and uterine microbiota. A total of 733 bacterial isolates (512 vaginal and 221 uterine isolates) were recovered from four different agar media under anaerobic and aerobic culturing conditions. Overall, the culturable fraction of the vaginal and uterine microbiota was noticeably different from that identified by 16S rRNA gene sequencing. Whereas Firmicutes (58%), Proteobacteria (28%), and Actinobacteria (12%) were the most frequently identified phyla among the cultured bacterial isolates, 16S rRNA gene sequencing indicated that 95% of the vaginal microbiota was composed of members from the Actinobacteria and Firmicutes in almost equal proportions. None of the prevalent genera (*Streptococcus*, *Bacillus*, *Escherichia*, and *Staphylococcus*) identified by vaginal culturing were among the 20 most relatively abundant genera in the vagina microbiota based on 16S rRNA gene sequencing. The most abundant genera in the uterine microbiota were also vastly different between culturing and sequencing. For example, we did not recover any species within the *Leptotrichiaceae*, *Oscillospiraceae*, or *Lachnospiraceae* families or *Fusobacterium* and *Prevotella* genera by culturing (Table S4). Thus, it highlights the need for culturing with specialized growth media supporting a wider range of bacteria under conditions that can better replicate the physiological and mucosal properties of the bovine reproductive tract. Another factor that may have limited the recovery of some bacterial species from the vaginal and uterine swabs was the freezing of the samples prior to culturing. It has been reported that freezer storage can influence viable bacterial cell recovery and that different species may tolerate freezing and the cryoprotectant differently (30, 65).

To the best of our knowledge, this is the first study to characterize the culturable portion of the bovine female reproductive microbiota. One of the more interesting and important findings from the culture results is that both the vagina and uterus in healthy cattle harbor bacterial species associated with disease. These bacteria species included *H. somni* (bovine respiratory disease) (66) *Moraxella bovis* (pinkeye) (67) *Moraxella bovoculi* (pinkeye) (67) ( *Staphylococcus aureus* (mastitis) (68) and *Trueperella abortisuis* (abortion) (69) (Table S4). Isolates from genera such as *Shigella*, *Streptococcus*, and *Mannheimia* that contain pathogenic species were also identified. The presence of these potentially pathogenic bacteria within the vagina and uterus begs the question of whether these bacteria are transferred to the offspring during birth or to bulls during natural breeding (70).

Antimicrobial resistance is a serious issue in both animal and public health, particularly with regard to dissemination through bovine-associated pathogenic and commensal bacteria (71). Therefore, surveillance of the resistome (the collection of AMR genes associated with the microbial community of a particular environment) is important for mitigating the emergence and spread of AMR (72). The microbial continuum along the female reproductive tract is an important source of seeding for the offspring calf (64, 70, 73) and bull reproductive tract microbiota (70). Considering that bacteria may be transferred from the female reproductive tract to the bull, then to other females, and ultimately to their offspring (59, 70), it is critical to investigate AMR in commensal bacteria present in the female reproductive tract of cattle. Our AST results for 29 aerobic vaginal and uterine isolates revealed that only one *E. coli* isolate exhibited phenotypic resistance with the majority of isolates susceptible to all the antimicrobials tested, suggesting that the female urogenital tract harbors fewer antimicrobial-resistant bacteria than anticipated. However, because breakpoints were not available for 21 of the antibiotics tested (five antibiotics in the Gram-positive panels and 16 antibiotics in Gram-negative panels), our AST results need to be interpreted cautiously. A larger set of bacterial isolates with bovine-specific antibiotic panels and, if possible, shotgun metagenomic sequencing would be needed to fully determine the scope of the resistome associated with the female reproductive microbiome.

### Conclusions

Although the structure, richness, and diversity of the vaginal microbiota did not differ in heifers that failed to become pregnant via AI compared with heifers that did, certain taxa were found to be associated with fertility. Twenty-eight ASVs were differentially abundant in the uterine microbiota between the two groups of cows, including *Methanobrevibacter ruminantium* and *F. necrophorum* which were positively associated with fertility. Our culturing results revealed that a relatively diverse assortment of bacterial species, mostly comprised of aerobic and anaerobic Gram-positive bacteria, is present in the bovine female reproductive tract. Most of the vaginal and uterine bacterial isolates screened did not show phenotypic antimicrobial resistance. Our identification of fertility-associated vaginal and uterine microbial taxa at the time of AI breeding highlights that it may be possible to develop reproductive microbiome-targeted strategies to enhance fertility in beef cattle.

## Data Availability

Raw sequence data are available from the NCBI Sequence Read Archive under BioProject accession PRJNA976303. All other data supporting the findings of this study are presented within the article.

## SUPPLEMENTAL MATERIAL

Supplemental material spectrum.02732-23-s0001.docx Tables S1 to S6.

ASM does not own the copyrights to Supplemental Material that may be linked to, or accessed through, an article. The authors have granted ASM a non-exclusive, world-wide license to publish the Supplemental Material files. Please contact the corresponding author directly for reuse.
