# Supplementary material for: Sequencing and culture-based characterization of the vaginal and uterine microbiota in beef cattle that became pregnant or remained open following artificial insemination
Source: Microbiol Spectr. 2023 Nov 3;11(6):e02732-23. doi: 10.1128/spectrum.02732-23 (PMC10714821; doi:10.1128/spectrum.02732-23)
Supplement: Supplemental material — Tables S1 to S6. [file spectrum.02732-23-s0001.docx]

**Supplementary Tables**

**Supplementary Table S1.** Percent relative abundance (± standard error of the mean) of the 20 most relatively abundant genera in the vaginal microbiota of heifers that became pregnant or remained open following artificial insemination.

| **Genus** | **Open** | **Pregnant** |
| --- | --- | --- |
| *Trueperella* | 52.73 ± 2.82 | 51.74 ± 2.97 |
| *Helcococcus* | 23.18 ± 2.05 | 26.59 ± 2.89 |
| *Peptoniphilus* | 12.16 ± 1.47 | 10.84 ± 1.36 |
| *Fusobacterium* | 0.31 ± 0.09 | 2.93 ± 1.65 |
| *Caviibacter* | 0.14 ± 0.07 | 1.61 ± 1.25 |
| *Oscillospiraceae* UCG-005 | 1.18 ± 0.39 | 0.38 ± 0.07 |
| *Corynebacterium* | 0.55 ± 0.2 | 0.38 ± 0.08 |
| *Romboutsia* | 0.49 ± 0.18 | 0.2 ± 0.04 |
| *Arcanobacterium* | 0.2 ± 0.05 | 0.49 ± 0.28 |
| *Christensenellaceae* R-7 group | 0.4 ± 0.16 | 0.14 ± 0.03 |
| *Streptobacillus* | 0.38 ± 0.25 | 0.11 ± 0.04 |
| *Clostridium* sensu stricto 1 | 0.27 ± 0.12 | 0.17 ± 0.04 |
| *Histophilus* | 0.19 ± 0.02 | 0.15 ± 0.02 |
| *Parvimonas* | 0.1 ± 0.04 | 0.22 ± 0.13 |
| *Alistipes* | 0.24 ± 0.13 | 0.04 ± 0.01 |
| *Campylobacter* | 0.07 ± 0.04 | 0.22 ± 0.14 |
| *Mannheimia* | 0.13 ± 0.02 | 0.15 ± 0.03 |
| *Rikenellaceae* RC9 gut group | 0.19 ± 0.13 | 0.04 ± 0.02 |
| *Monoglobus* | 0.17 ± 0.07 | 0.04 ± 0.01 |
| *Mycoplasma* | 0.1 ± 0.01 | 0.09 ± 0.01 |

**Supplementary Table S2**. Percent relative abundance (± standard error of the mean) of the 20 most relatively abundant genera in the vaginal microbiota of cows that became pregnant or remained open following artificial insemination.

| **Genus** | **Open** | **Pregnant** |
| --- | --- | --- |
| *Oscillospiraceae* UCG-005 | 13.95 ± 0.37 | 13.67 ± 0.48 |
| *Rikenellaceae* RC9 gut group | 4.95 ± 0.23 | 4.44 ± 0.19 |
| *Christensenellaceae* R-7 group | 3.94 ± 0.13 | 3.57 ± 0.15 |
| *Alistipes* | 3.33 ± 0.13 | 3.1 ± 0.12 |
| *Bacteroides* | 3.25 ± 0.15 | 3.12 ± 0.12 |
| *Streptobacillus* | 1.7 ± 0.73 | 4.34 ± 1.44 |
| *Corynebacterium* | 2.85 ± 0.9 | 1.95 ± 0.46 |
| *Romboutsia* | 2.27 ± 0.14 | 2.3 ± 0.11 |
| *Monoglobus* | 2.07 ± 0.07 | 1.83 ± 0.08 |
| *Oscillospiraceae* NK4A214 group | 1.65 ± 0.07 | 1.56 ± 0.07 |
| *Prevotellaceae* UCG-004 | 1.35 ± 0.06 | 1.25 ± 0.06 |
| P*revotellaceae* UCG-003 | 1.29 ± 0.05 | 1.2 ± 0.06 |
| *Leptotrichia* | 0.98 ± 0.53 | 1.32 ± 0.57 |
| *Butyricicoccaceae* UCG-009 | 1.19 ± 0.03 | 1.15 ± 0.04 |
| *Anaerovoracaceae* Family XIII AD3011 group | 1.18 ± 0.03 | 1.15 ± 0.05 |
| *Akkermansia* | 1.13 ± 0.16 | 1.13 ± 0.15 |
| *Dorea* | 0.91 ± 0.04 | 0.98 ± 0.04 |
| *Rikenellaceae* dgA-11 gut group | 0.83 ± 0.04 | 0.82 ± 0.04 |
| *Lachnospiraceae UCG-010* | 0.6 ± 0.02 | 0.61 ± 0.02 |
| *Coprococcus* | 0.55 ± 0.02 | 0.59 ± 0.03 |

**Supplementary Table S3.** Percent relative abundance (± standard error of the mean) of the 20 most relatively abundant genera in the uterine microbiota of cows that became pregnant or remained open following artificial insemination.

| **Genus** | **Open** | **Pregnant** |
| --- | --- | --- |
| *Oscillospiraceae* UCG-005 | 13.69 ± 0.72 | 12.91 ± 0.63 |
| *Christensenellaceae* R-7 group | 3.23 ± 0.25 | 3.4 ± 0.29 |
| *Helcococcus* | 1.52 ± 0.68 | 4.4 ± 1.26 |
| *Romboutsia* | 2.99 ± 0.38 | 2.7 ± 0.27 |
| *Rikenellaceae* RC9 gut group | 2.74 ± 0.32 | 2.89 ± 0.29 |
| *Trueperella* | 1.96 ± 0.81 | 3.27 ± 0.85 |
| *Alistipes* | 2.29 ± 0.21 | 2.08 ± 0.17 |
| *Bacteroides* | 2.34 ± 0.35 | 1.91 ± 0.17 |
| *Clostridium* sensu stricto 7 | 1.68 ± 0.42 | 2.45 ± 0.39 |
| *Oscillospiraceae* NK4A214 group | 1.65 ± 0.21 | 1.42 ± 0.1 |
| *Corynebacterium* | 1.86 ± 0.53 | 1.21 ± 0.35 |
| *Prevotellaceae* UCG-003 | 1.09 ± 0.15 | 1.68 ± 0.46 |
| *Monoglobus* | 1.52 ± 0.13 | 1.18 ± 0.11 |
| *Anaerovoracaceae* Family XIII AD3011 group | 1.12 ± 0.11 | 1.08 ± 0.09 |
| *Prevotella* | 1.33 ± 0.51 | 0.8 ± 0.37 |
| *Peptoniphilus* | 0.76 ± 0.34 | 1.18 ± 0.4 |
| Prevotellaceae UCG-004 | 1.22 ± 0.2 | 0.71 ± 0.08 |
| *Butyricicoccaceae* UCG-009 | 1 ± 0.19 | 0.85 ± 0.07 |
| *Dorea* | 0.83 ± 0.09 | 0.8 ± 0.07 |
| *Lachnospiraceae* NK3A20 group | 1.16 ± 0.57 | 0.23 ± 0.06 |

**Supplemental Table S4.** A list of all bacterial isolates recovered, their growth media and oxygen condition, and their anatomic origin.

| **Phylum** | **Bacteria Name** | **Number of Isolates** | | **Isolate Origin** | | **Media** | | |
| --- | --- | --- | --- | --- | --- | --- | --- | --- |
|  |  | **Aerobic** | **Anaerobic** | **Uterus** | **Vagina** | **CB/Blood** | **MRS** | **WC** |
| Firmicutes | *Abyssicoccus albus* | - | 1 | - | 1 | - | - | 1 |
| Proteobacteria | *Acinetobacter lwoffii* | 3 | - | - | 3 | 3 | - | - |
| Proteobacteria | *Acinetobacter seohaensis* | 2 | - | - | 2 | 2 | - | - |
| Proteobacteria | *Actinobacillus seminis* | 3 | 9 | 5 | 7 | 4 | - | 8 |
| Actinobacteria | *Actinomadura formosensis* | 1 | - | - | 1 | - | 1 | - |
| Firmicutes | *Aerococcus sanguinicola* | - | 1 | - | 1 | - | 1 | - |
| Firmicutes | *Aerococcus urinaeequi* | 2 | 2 | - | 4 | 1 | 2 | 1 |
| Firmicutes | *Aerococcus urinaehominis* | - | 3 | - | 3 | - | - | 3 |
| Firmicutes | *Aerococcus viridans* | 3 | 5 | 2 | 6 | 5 | 1 | 2 |
| Firmicutes | *Alkalihalobacillus clausii* | 5 | - | - | 5 | 5 | - | - |
| Actinobacteria | *Arcanobacterium wilhelmae* | - | 1 | 1 | - | - | 1 | - |
| Actinobacteria | *Arthrobacter gandavensis* | 1 | - | - | 1 | 1 | - | - |
| Actinobacteria | *Arthrobacter koreensis* | 1 | - | - | 1 | 1 | - | - |
| Proteobacteria | *Atlantibacter hermannii* | - | 1 | - | 1 | - | 1 | - |
| Firmicutes | *Bacillus aerius* | 3 | - | 2 | 1 | 2 | 1 | - |
| Firmicutes | *Bacillus atrophaeus* | 1 | - | - | 1 | - | 1 | - |
| Firmicutes | *Bacillus australimaris* | 1 | - | - | 1 | 1 | - | - |
| Firmicutes | *Bacillus cereus* | 1 | - | 1 | - | 1 | - | - |
| Firmicutes | *Bacillus gobiensis* | 1 | - | - | 1 | - | 1 | - |
| Firmicutes | *Bacillus haynesii* | 12 | - | 2 | 10 | 5 | 7 | - |
| Firmicutes | *Bacillus licheniformis* | 4 | 7 | 1 | 10 | 8 | 2 | 1 |
| Firmicutes | *Bacillus nakamurai* | 1 | - | - | 1 | - | 1 | - |
| Firmicutes | *Bacillus pacificus* | - | 2 | - | 2 | - | - | 2 |
| Firmicutes | *Bacillus paralicheniformis* | 30 | 20 | 9 | 41 | 19 | 20 | 11 |
| Firmicutes | *Bacillus paramobilis* | 1 | - | 1 | - | - | 1 | - |
| Firmicutes | *Bacillus piscis* | 4 | - | - | 4 | 1 | 3 | - |
| Firmicutes | *Bacillus proteolyticus* | - | 2 | - | 2 | - | 1 | 1 |
| Firmicutes | *Bacillus pumilus* | 35 | - | 15 | 20 | 27 | 8 | - |
| Firmicutes | *Bacillus sonorensis* | 2 | - | 1 | 1 | 1 | 1 | - |
| Firmicutes | *Bacillus stratosphericus* | 1 | - | - | 1 | - | 1 | - |
| Firmicutes | *Bacillus tropicus* | - | 1 | - | 1 | - | - | 1 |
| Firmicutes | *Bacillus wiedmannii* | 1 | - | 1 | - | 1 | - | - |
| Firmicutes | *Bacillus xiamenensis* | 1 | - | - | 1 | - | 1 | - |
| Firmicutes | *Bacillus zhangshouensis* | 1 | - | 1 | - | - | 1 | - |
| Firmicutes | *Brevibacillus borstelensis* | 1 | - | - | 1 | 1 | - | - |
| Firmicutes | *Caldibacillus hisashii* | - | 1 | 1 | - | - | 1 | - |
| Proteobacteria | *Campylobacter sputorum* | - | 1 | - | 1 | - | - | 1 |
| Firmicutes | *Clostridium saccharoperbutylacetonicum* | - | 1 | - | 1 | - | - | 1 |
| Actinobacteria | *Corynebacterium confusum* | 1 | - | - | 1 | 1 | - | - |
| Actinobacteria | *Corynebacterium lactis* | 3 | - | - | 3 | 3 | - | - |
| Actinobacteria | *Corynebacterium renale* | 11 | 16 | 9 | 18 | 15 | 1 | 11 |
| Actinobacteria | *Corynebacterium vitaeruminis* | 1 | - | - | 1 | 1 | - | - |
| Actinobacteria | *Corynebacterium xerosis* | 2 | - | - | 2 | 2 | - | - |

**Supplemental Table 4 Cont’d.** A list of all bacterial isolates recovered, their growth media and oxygen condition, and their anatomic origin.

| **Phylum** | **Bacteria Name** | **Number of Isolates** | | **Isolate Origin** | | **Media** | | |
| --- | --- | --- | --- | --- | --- | --- | --- | --- |
|  |  | **Aerobic** | **Anaerobic** | **Uterus** | **Vagina** | **CB/Blood** | **MRS** | **WC** |
| Deferribacteres | *Denitrovibrio acetiphilus* | - | 1 | - | 1 | - | - | 1 |
| Firmicutes | *Enterococcus aquimarinus* | - | 1 | 1 | - | 1 | - | - |
| Firmicutes | *Enterococcus casseliflavus* | - | 1 | - | 1 | - | - | 1 |
| Firmicutes | *Enterococcus gallinarum* | - | 3 | - | 3 | 1 | 2 | - |
| Firmicutes | *Enterococcus hirae* | - | 2 | 1 | 1 | - | 2 | - |
| Firmicutes | *Erysipelothrix inopinata* | - | 1 | 1 | - | 1 | - | - |
| Proteobacteria | *Escherichia coli* | - | 1 | - | 1 | - | 1 | - |
| Proteobacteria | *Escherichia fergusonii* | - | 11 | 4 | 7 | 1 | 6 | 4 |
| Proteobacteria | *Escherichia fergusonii* ATCC 35469 | 25 | 1 | 9 | 17 | 16 | 10 | - |
| Proteobacteria | *Escherichia marmotae* | 9 | 1 | 1 | 9 | 6 | 4 | - |
| Firmicutes | *Facklamia hominis* | 4 | 8 | 6 | 6 | 8 | 3 | 1 |
| Proteobacteria | *Gallibacterium salpingitidis* | 2 | 2 | 4 | - | 2 | 1 | 1 |
| Firmicutes | *Helcococcus ovis* | 1 | - | - | 1 | 1 | - | - |
| Firmicutes | *Heyndrickxia oleronia* | 5 | - | - | 5 | 1 | 4 | - |
| Proteobacteria | *Histophilus somni* | 2 | - | 1 | 1 | 2 | - | - |
| Firmicutes | *Hutsoniella sourekii* | 2 | 4 | - | 6 | 3 | - | 3 |
| Proteobacteria | *Hydrogenophaga electricum* | 1 | - | - | 1 | 1 | - | - |
| Proteobacteria | *Kosakonia oryziphila* | 1 | - | - | 1 | - | 1 | - |
| Actinobacteria | *Kribbella monticola* | - | 1 | 1 | - | 1 | - | - |
| Firmicutes | *Kurthia gibsonii* | - | 1 | - | 1 | - | - | 1 |
| Firmicutes | *Lacrimispora algidixylanolytica* | - | 1 | - | 1 | - | 1 | - |
| Firmicutes | *Lacticaseibacillus pantheris* | 1 | - | 1 | - | 1 | - | - |
| Firmicutes | *Lactiplantibacillus plantarum* | - | 1 | - | 1 | - | 1 | - |
| Firmicutes | *Lactobacillus amylovorus* | - | 2 | - | 2 | 1 | 1 | - |
| Proteobacteria | *Lelliottia amnigena* | - | 1 | - | 1 | - | - | 1 |
| Firmicutes | *Limosilactobacillus mucosae* | - | 5 | 4 | 1 | 1 | 4 | - |
| Firmicutes | *Lysinibacillus macroides* | 2 | - | - | 2 | 1 | 1 | - |
| Firmicutes | *Lysinibacillus pakistanensis* | 1 | - | - | 1 | - | 1 | - |
| Firmicutes | *Macrococcus canis* | 4 | - | 1 | 3 | 4 | - | - |
| Proteobacteria | *Mannheimia granulomatis* | - | 1 | - | 1 | - | - | 1 |
| Proteobacteria | *Mannheimia ruminalis* | 2 | - | - | 2 | 2 | - | - |
| Proteobacteria | *Mannheimia varigena* | 1 | 3 | 1 | 3 | 1 | - | 3 |
| Proteobacteria | *Moraxella bovis* | 1 | - | - | 1 | 1 | - | - |
| Proteobacteria | *Moraxella bovoculi* | 2 | - | 2 | - | 2 | - | - |
| Proteobacteria | *Moraxella catarrhalis* | 1 | - | - | 1 | 1 | - | - |
| Proteobacteria | *Moraxella osloensis* | 3 | - | 2 | 1 | 3 | - | - |
| Proteobacteria | *Neisseria weaveri* | - | 1 | 1 | - | - | - | 1 |
| Firmicutes | *Neobacillus cucumis* | - | 1 | - | 1 | 1 | - | - |
| Firmicutes | *Niallia circulans* | 3 | 1 | 1 | 3 | 3 | 1 | - |
| Firmicutes | *Paenibacillus borealis* | - | 1 | - | 1 | - | - | 1 |
| Firmicutes | *Paenibacillus campinasensis* | 1 | - | - | 1 | 1 | - | - |
| Firmicutes | *Paenibacillus daejeonensis* | 1 | - | 1 | - | 1 | - | - |
| Firmicutes | *Paenibacillus lautus* | 1 | - | - | 1 | 1 | - | - |

**Supplemental Table 4 Cont’d.** A list of all bacterial isolates recovered, their growth media and oxygen condition, and their anatomic origin.

| **Phylum** | **Bacteria Name** | **Number of Isolates** | | **Isolate Origin** | | **Media** | | |
| --- | --- | --- | --- | --- | --- | --- | --- | --- |
|  |  | **Aerobic** | **Anaerobic** | **Uterus** | **Vagina** | **CB/Blood** | **MRS** | **WC** |
| Firmicutes | *Paenibacillus macerans* | - | 1 | - | 1 | - | - | 1 |
| Firmicutes | *Paenibacillus mobilis* | - | 1 | - | 1 | - | - | 1 |
| Firmicutes | *Paenibacillus naphthalenovorans* | 5 | - | - | 5 | 5 | - | - |
| Firmicutes | *Paenibacillus polysaccharolyticus* | - | 1 | - | 1 | 1 | - | - |
| Firmicutes | *Paenibacillus silvae* | 1 | - | - | 1 | 1 | - | - |
| Proteobacteria | *Pelistega europaea* | 1 | - | 1 | - | 1 | - | - |
| Firmicutes | *Peptoniphilus indolicus* | - | 7 | - | 7 | 4 | 1 | 2 |
| Firmicutes | *Peribacillus psychrosaccharolyticus* | - | 1 | - | 1 | - | - | 1 |
| Firmicutes | *Periweissella fabalis* | - | 1 | - | 1 | - | - | 1 |
| Firmicutes | *Priestia aryabhattai* | 1 | - | 1 | - | - | 1 | - |
| Firmicutes | *Priestia megaterium* | 2 | - | 1 | 1 | 1 | 1 | - |
| Proteobacteria | *Proteus mirabilis* | 1 | 9 | - | 10 | 6 | 1 | 3 |
| Proteobacteria | *Pseudescherichia vulneris* | 1 | - | - | 1 | - | 1 | - |
| Proteobacteria | *Pseudomonas flexibilis* | 2 | - | 2 | - | 2 | - | - |
| Firmicutes | *Robertmurraya siralis* | 1 | - | - | 1 | 1 | - | - |
| Firmicutes | *Rossellomorea marisflavi* | 1 | - | - | 1 | - | 1 | - |
| Actinobacteria | *Rothia nasimurium* | 1 | - | - | 1 | 1 | - | - |
| Proteobacteria | *Shigella boydii* | 1 | - | - | 1 | 1 | - | - |
| Proteobacteria | *Shigella flexneri* | 1 | 1 | 1 | 1 | 1 | 1 | = |
| Proteobacteria | *Shigella sonnei* | - | 1 | 1 | - | - | - | 1 |
| Firmicutes | *Solibacillus isronensis* | 1 | - | 1 | - | 1 | - | - |
| Firmicutes | *Staphylococcus aureus* | - | 1 | - | 1 | - | 1 | - |
| Firmicutes | *Staphylococcus capitis* | 2 | - | 2 | - | 2 | - | - |
| Firmicutes | *Staphylococcus chromogenes* | 6 | 11 | 2 | 15 | 9 | 1 | 7 |
| Firmicutes | *Staphylococcus epidermidis* | 1 | 1 | - | 2 | 2 | - | - |
| Firmicutes | *Staphylococcus hominis* | - | 1 | - | 1 | 1 | - | - |
| Firmicutes | *Staphylococcus hyicus* | 6 | - | 1 | 5 | 5 | 1 | - |
| Firmicutes | *Staphylococcus pasteuri* | - | 1 | - | 1 | - | 1 | - |
| Firmicutes | *Staphylococcus roterodami* | 1 | - | - | 1 | 1 | - | - |
| Firmicutes | *Streptococcus anginosus* | - | 1 | - | 1 | - | - | 1 |
| Firmicutes | *Streptococcus equinus* | 2 | 38 | 9 | 31 | 13 | 16 | 11 |
| Firmicutes | *Streptococcus equinus* ATCC 9812 | 8 | - | 7 | 1 | 2 | 6 | - |
| Firmicutes | *Sterptococcus lutetiensis* | 4 | 5 | 2 | 7 | 3 | 5 | 1 |
| Firmicutes | *Streptococcus pluranimalium* | 82 | 151 | 88 | 145 | 117 | 83 | 33 |
| Firmicutes | *Streptococcus ruminantium* | 1 | - | - | 1 | 1 | - | - |
| Firmicutes | *Streptococcus suis* | 1 | 3 | 1 | 3 | 1 | - | 3 |
| Actinobacteria | *Streptomyces viridodiastaticus* | 1 | - | - | 1 | 1 | - | - |
| Proteobacteria | *Thioalkalivibrio jannaschii* | - | 1 | - | 1 | - | - | 1 |
| Actinobacteria | *Trueperella abortisuis* | 12 | - | 1 | 11 | 12 | - | - |
| Firmicutes | *Weizmannia coagulans* | - | 2 | - | 2 | - | 2 | - |
| **Total number of isolates** | | ***363*** | ***370*** | ***216*** | ***517*** | ***377*** | ***226*** | ***130*** |

**Supplementary Table 5.** Antimicrobial resistance breakpoints that have been reported previously (Gram-positive) (44-46).^1^

| Antibiotic | *Corynebacterium renale* (14121.US_CB-D) | *Staphylococcus chromogenes* (19197.US_CB-C) | *Staphylococcus chromogenes* (18207.US_CB-D) | *Corynebacterium renale* (14137.CB-1.VS) | *Emterococcus hirae* (17168.V-An_MRS-A) | *Staphylococcus aureus* (14177.V-An_MRS-A) | *Staphylococcus chromogenes* (247_CB-C.VS) | *Staphylococcus chromogenes* (240_CB-B.VS) | *Staphylococcus chromogenes* (306V_CB-A) | *Staphylococcus epidermidis* (101.CB-6.VS) |
| --- | --- | --- | --- | --- | --- | --- | --- | --- | --- | --- |
| Amikacin | ? | ? | ? | ? | - | ? | ? | ? | ? | ? |
| Ampicillin | ≤ 0.12/0.25-2/≥ 4 | ? | ? | ≤ 0.12/0.25-2/≥ 4 | ? | ? | ? | ? | ? | ? |
| Amoxicillin-clavulanate | ≤ 0.12/0.25-2/≥ 4 | ≤ 2/-/≥ 4 | ≤ 2/-/≥ 4 | ≤ 0.12/0.25-2/≥ 4 | ≤ 8/-/≥ 16 | ≤ 2/-/≥ 4 | ≤ 2/-/≥ 4 | ≤ 2/-/≥ 4 | ≤ 2/-/≥ 4 | ≤ 2/-/≥ 4 |
| Cephalothin | ? | ≤ 2/-/≥ 4 | ≤ 2/-/≥ 4 | ? | - | ≤ 2/-/≥ 4 | ≤ 2/-/≥ 4 | ≤ 2/-/≥ 4 | ≤ 2/-/≥ 4 | ≤ 2/-/≥ 4 |
| Clindamycin | ≤ 0.5/1-2/≥ 4 | ≤ 0.5/1-2/≥ 4 | ≤ 0.5/1-2/≥ 4 | ≤ 0.5/1-2/≥ 4 | - | ≤ 0.5/1-2/≥ 4 | ≤ 0.5/1-2/≥ 4 | ≤ 0.5/1-2/≥ 4 | ≤ 0.5/1-2/≥ 4 | ≤ 0.5/1-2/≥ 4 |
| Cefazolin | ? | ≤ 2/-/≥ 4 | ≤ 2/-/≥ 4 | ? | - | ≤ 2/-/≥ 4 | ≤ 2/-/≥ 4 | ≤ 2/-/≥ 4 | ≤ 2/-/≥ 4 | ≤ 2/-/≥ 4 |
| Gentamicin | ≤ 4/8/≥ 16 | ≤ 4/8/≥ 16 | ≤ 4/8/≥ 16 | ≤ 4/8/≥ 16 | - | ≤ 4/8/≥ 16 | ≤ 4/8/≥ 16 | ≤ 4/8/≥ 16 | ≤ 4/8/≥ 16 | ≤ 4/8/≥ 16 |
| Trimethoprim-sulfamethoxazole | ≤ 2/38/-/≥ 4/76 | ≤ 2/-/≥ 4 | ≤ 2/-/≥ 4 | ≤ 2/38/-/≥ 4/76 | - | ≤ 2/-/≥ 4 | ≤ 2/-/≥ 4 | ≤ 2/-/≥ 4 | ≤ 2/-/≥ 4 | ≤ 2/-/≥ 4 |
| Minocycline | ? | ≤ 4/8/≥ 16 | ≤ 4/8/≥ 16 | ? | ≤ 4/8/≥ 16 | ≤ 4/8/≥ 16 | ≤ 4/8/≥ 16 | ≤ 4/8/≥ 16 | ≤ 4/8/≥ 16 | ≤ 4/8/≥ 16 |
| Chloramphenicol | ? | ≤ 8/16/≥ 32 | ≤ 8/16/≥ 32 | ? | ≤ 8/16/≥ 32 | ≤ 8/16/≥ 32 | ≤ 8/16/≥ 32 | ≤ 8/16/≥ 32 | ≤ 8/16/≥ 32 | ≤ 8/16/≥ 32 |
| Marbofloxacin | ? | ? | ? | ? | ? | ? | ? | ? | ? | ? |
| Erythromycin | ≤ 0.5/1/≥ 2 | ≤ 0.5/1-4/≥ 8 | ≤ 0.5/1-4/≥ 8 | ≤ 0.5/1/≥ 2 | ≤ 0.5/1-4/≥ 8 | ≤ 0.5/1-4/≥ 8 | ≤ 0.5/1-4/≥ 8 | ≤ 0.5/1-4/≥ 8 | ≤ 0.5/1-4/≥ 8 | ≤ 0.5/1-4/≥ 8 |
| Pradofloxacin | ? | ? | ? | ? | ? | ? | ? | ? | ? | ? |
| Penicillin G | ≤ 0.12/0.25-2/≥ 4 | ≤ 0.12/-/≥ 0.25 | ≤ 0.12/-/≥ 0.25 | ≤ 0.12/0.25-2/≥ 4 | ≤ 8/-/≥ 16 | ≤ 0.12/-/≥ 0.25 | ≤ 0.12/-/≥ 0.25 | ≤ 0.12/-/≥ 0.25 | ≤ 0.12/-/≥ 0.25 | ≤ 0.12/-/≥ 0.25 |
| Enrofloxacin | ? | ? | ? | ? | ? | ? | ? | ? | ? | ? |
| Nitrofurantoin | ? | ≤ 32/64/≥ 128 | ≤ 32/64/≥ 128 | ? | ≤ 32/64/≥ 128 | ≤ 32/64/≥ 128 | ≤ 32/64/≥ 128 | ≤ 32/64/≥ 128 | ≤ 32/64/≥ 128 | ≤ 32/64/≥ 128 |
| Imipenem | ? | ? | ? | ? | ≤ 0.001/-/≥ 4 | ? | ? | ? | ? | ? |
| Doxycycline | ≤ 4/8/≥ 16 | ≤ 4/8/≥ 16 | ≤ 4/8/≥ 16 | ≤ 4/8/≥ 16 | ≤ 4/8/≥ 16 | ≤ 4/8/≥ 16 | ≤ 4/8/≥ 16 | ≤ 4/8/≥ 16 | ≤ 4/8/≥ 16 | ≤ 4/8/≥ 16 |
| Cefpodoxime | ? | ≤ 2/-/≥ 4 | ≤ 2/-/≥ 4 | ? | ? | ≤ 2/-/≥ 4 | ≤ 2/-/≥ 4 | ≤ 2/-/≥ 4 | ≤ 2/-/≥ 4 | ≤ 2/-/≥ 4 |
| Tetracycline | ≤ 4/8/≥ 16 | ≤ 4/8/≥ 16 | ≤ 4/8/≥ 16 | ≤ 4/8/≥ 16 | ≤ 4/8/≥ 16 | ≤ 4/8/≥ 16 | ≤ 4/8/≥ 16 | ≤ 4/8/≥ 16 | ≤ 4/8/≥ 16 | ≤ 4/8/≥ 16 |
| Cefovecin | ? | ≤ 2/-/≥ 4 | ≤ 2/-/≥ 4 | ? | ? | ≤ 2/-/≥ 4 | ≤ 2/-/≥ 4 | ≤ 2/-/≥ 4 | ≤ 2/-/≥ 4 | ≤ 2/-/≥ 4 |
| Vancomycin | ≤ 2/-/- | ≤ 4/8-16/≥ 32 | ≤ 4/8-16/≥ 32 | ≤ 2/-/- | ≤ 4/8-16/≥ 32 | ≤ 4/8-16/≥ 32 | ≤ 4/8-16/≥ 32 | ≤ 4/8-16/≥ 32 | ≤ 4/8-16/≥ 32 | ≤ 4/8-16/≥ 32 |
| Oxacillin | ? | ≤ 2/-/≥ 4 | ≤ 2/-/≥ 4 | ? | ? | ≤ 2/-/≥ 4 | ≤ 2/-/≥ 4 | ≤ 2/-/≥ 4 | ≤ 2/-/≥ 4 | ≤ 2/-/≥ 4 |
| Rifampin | ≤ 1/2/≥ 4 | ≤ 1/2/≥ 4 | ≤ 1/2/≥ 4 | ≤ 1/2/≥ 4 | ≤ 1/2/≥ 4 | ≤ 1/2/≥ 4 | ≤ 1/2/≥ 4 | ≤ 1/2/≥ 4 | ≤ 1/2/≥ 4 | ≤ 1/2/≥ 4 |

^1^MIC values (µg/ml) presented in the table were obtained from the Sensititre Gram Positive Companion panel (COMPGP1F).

GREEN= no break points (isolate)

BLUE= no/few break points (antibiotic)

RED= resistant

DARK RED/WHITE TEXT= intrinsically resistant

YELLOW= highest test concentrations were lower than breakpoint

ORANGE= intermediate

**Supplementary Table 6**. Antimicrobial resistance breakpoints that have been reported previously (Gram-negative) (44-46, 81, 82)^1^.

| Antibiotic | *Escherichia coli* (18207.US_CB-C) | *Escherichia coli* (14121.US_MRS-B) | *Escherichia coli* (18333.US_CB-C) | *Histophilus somni* (20116.US_CB-A) | *Actinobacillus seminist* (306V_CB-B) | *Escherichia coli* (169_CB-A.VS) | *Escherichia coli* (7091.V-An_Bld-A) | *Escherichia coli* (18081.CB-1.VS) | *Escherichia coli* (247_CB-D.VS) | *Escherichia coli* (247_MRS-A.VS) |
| --- | --- | --- | --- | --- | --- | --- | --- | --- | --- | --- |
| Amikacin | ≤ 16/32/≥ 64 | ≤ 16/32/≥ 64 | ≤ 16/32/≥ 64 | - | - | ≤ 16/32/≥ 64 | ≤ 16/32/≥ 64 | ≤ 16/32/≥ 64 | ≤ 16/32/≥ 64 | ≤ 16/32/≥ 64 |
| Ampicillin | ≤ 8/16/≥ 32 | ≤ 8/16/≥ 32 | ≤ 8/16/≥ 32 | ? | ? | ≤ 8/16/≥ 32 | ≤ 8/16/≥ 32 | ≤ 8/16/≥ 32 | ≤ 8/16/≥ 32 | ≤ 8/16/≥ 32 |
| Azithromycin | ≤ 16/-/≥ 32 | ≤ 16/-/≥ 32 | ≤ 16/-/≥ 32 | - | - | ≤ 16/-/≥ 32 | ≤ 16/-/≥ 32 | ≤ 16/-/≥ 32 | ≤ 16/-/≥ 32 | ≤ 16/-/≥ 32 |
| Ceftiofur | - | - | - | ≤ 2/-/≥ 8 | ? | - | - | - | - | - |
| Clindamycin | - | - | - | ? | ? | - | - | - | - | - |
| Danofloxacin | - | - | - | ? | ? | - | - | - | - | - |
| Cefazolin | ≤ 2/4/≥ 8 | ≤ 2/4/≥ 8 | ≤ 2/4/≥ 8 | - | - | ≤ 2/4/≥ 8 | ≤ 2/4/≥ 8 | ≤ 2/4/≥ 8 | ≤ 2/4/≥ 8 | ≤ 2/4/≥ 8 |
| Gentamicin | ≤ 4/8/≥ 16 | ≤ 4/8/≥ 16 | ≤ 4/8/≥ 16 | ? | ? | ≤ 4/8/≥ 16 | ≤ 4/8/≥ 16 | ≤ 4/8/≥ 16 | ≤ 4/8/≥ 16 | ≤ 4/8/≥ 16 |
| Gamithromycin | - | - | - | ? | ? | - | - | - | - | - |
| Trimethoprim-sulfamethoxazole | ≤ 2/-/≥ 4 | ≤ 2/-/≥ 4 | ≤ 2/-/≥ 4 | ? | ? | ≤ 2/-/≥ 4 | ≤ 2/-/≥ 4 | ≤ 2/-/≥ 4 | ≤ 2/-/≥ 4 | ≤ 2/-/≥ 4 |
| Ceftiofur | ≤ 2/4/≥ 8 | ≤ 2/4/≥ 8 | ≤ 2/4/≥ 8 | ? | - | ≤ 2/4/≥ 8 | ≤ 2/4/≥ 8 | ≤ 2/4/≥ 8 | ≤ 2/4/≥ 8 | ≤ 2/4/≥ 8 |
| Chloramphenicol | ≤ 8/16/≥ 32 | ≤ 8/16/≥ 32 | ≤ 8/16/≥ 32 | - | - | ≤ 8/16/≥ 32 | ≤ 8/16/≥ 32 | ≤ 8/16/≥ 32 | ≤ 8/16/≥ 32 | ≤ 8/16/≥ 32 |
| Ceftazidime | ≤ 4/8/≥ 16 | ≤ 4/8/≥ 16 | ≤ 4/8/≥ 16 | - | - | ≤ 4/8/≥ 16 | ≤ 4/8/≥ 16 | ≤ 4/8/≥ 16 | ≤ 4/8/≥ 16 | ≤ 4/8/≥ 16 |
| Erythromycin | ? | ? | ? | - | - | ? | ? | ? | ? | ? |
| Clarithromycin | ? | ? | ? | - | - | ? | ? | ? | ? | ? |
| Penicillin G | ? | ? | ? | ? | ? | ? | ? | ? | ? | ? |
| Enrofloxacin | ? | ? | ? | ≤ 0.25/-/≥ 2 | ? | ? | ? | ? | ? | ? |
| Imipenem | ≤ 1/2/≥ 4 | ≤ 1/2/≥ 4 | ≤ 1/2/≥ 4 | - | - | ≤ 1/2/≥ 4 | ≤ 1/2/≥ 4 | ≤ 1/2/≥ 4 | ≤ 1/2/≥ 4 | ≤ 1/2/≥ 4 |
| Doxycycline | ≤ 4/8/≥ 16 | ≤ 4/8/≥ 16 | ≤ 4/8/≥ 16 | - | - | ≤ 4/8/≥ 16 | ≤ 4/8/≥ 16 | ≤ 4/8/≥ 16 | ≤ 4/8/≥ 16 | ≤ 4/8/≥ 16 |
| Tetracycline | ≤ 4/8/≥ 16 | ≤ 4/8/≥ 16 | ≤ 4/8/≥ 16 | ≤ 2/-/≥ 8 | ? | ≤ 4/8/≥ 16 | ≤ 4/8/≥ 16 | ≤ 4/8/≥ 16 | ≤ 4/8/≥ 16 | ≤ 4/8/≥ 16 |
| Ticarcillin | ≤ 16/32-64/≥ 128 | ≤ 16/32-64/≥ 128 | ≤ 16/32-64/≥ 128 | - | - | ≤ 16/32-64/≥ 128 | ≤ 16/32-64/≥ 128 | ≤ 16/32-64/≥ 128 | ≤ 16/32-64/≥ 128 | ≤ 16/32-64/≥ 128 |
| Ticarcillin-clavulanate | ? | ? | ? | - | - | ? | ? | ? | ? | ? |
| Oxacillin | ? | ? | ? | - | - | ? | ? | ? | ? | ? |
| Rifampin | ? | ? | ? | - | - | ? | ? | ? | ? | ? |
| Sulfadimethoxine | - | - | - | ? | ? | - | - | - | - | - |
| Tiamulin | - | - | - | ? | ? | - | - | - | - | - |
| Tildipirosin | - | - | - | ? | ? | - | - | - | - | - |
| Tilmicosin | - | - | - | ≤ 8/-/≥ 32 | ? | - | - | - | - | - |
| Florfenicol | - | - | - | ≤ 16/-/≥ 64 | ? | - | - | - | - | - |
| Tulathromycin | - | - | - | ? | ? | - | - | - | - | - |
| Tylosin tartrate | - | - | - | ? | ? | - | - | - | - | - |
| Neomycin | - | - | - | ? | ? | - | - | - | - | - |
| Spectinomycin | - | - | - | ? | - | - | - | - | - | - |

**Supplementary Table 6 Cont’d**. Antimicrobial resistance breakpoints that have been reported previously (Gram-negative) (44-46, 81)^1^.

| Antibiotic | *Escherichia coli* (96_MRS-B.VS) | *Escherichia coli* (240_CB-A.VS) | *Escherichia coli* ( 46_CB-A.VS) | *Escherichia coli* (240_MRS-A.VS) | *Escherichia coli* (247_CB-B.VS) | *Escherichia coli* (267_CB-A.VS) | *Escherichia coli* (19044.V-An_MRS-A) | *Escherichia coli* (247_MRS-C.VS) | *Histophilus somni* (029V_CB-B) |
| --- | --- | --- | --- | --- | --- | --- | --- | --- | --- |
| Amikacin | ≤ 16/32/≥ 64 | ≤ 16/32/≥ 64 | ≤ 16/32/≥ 64 | ≤ 16/32/≥ 64 | ≤ 16/32/≥ 64 | ≤ 16/32/≥ 64 | ≤ 16/32/≥ 64 | ≤ 16/32/≥ 64 | - |
| Ampicillin | ≤ 8/16/≥ 32 | ≤ 8/16/≥ 32 | ≤ 8/16/≥ 32 | ≤ 8/16/≥ 32 | ≤ 8/16/≥ 32 | ≤ 8/16/≥ 32 | ≤ 8/16/≥ 32 | ≤ 8/16/≥ 32 | ? |
| Azithromycin | ≤ 16/-/≥ 32 | ≤ 16/-/≥ 32 | ≤ 16/-/≥ 32 | ≤ 16/-/≥ 32 | ≤ 16/-/≥ 32 | ≤ 16/-/≥ 32 | ≤ 16/-/≥ 32 | - | - |
| Ceftiofur | - | - | - | - | - | - | - | - | ≤ 2/-/≥ 8 |
| Clindamycin | - | - | - | - | - | - | - | ? | ? |
| Danofloxacin | - | - | - | - | - | - | - | - | ? |
| Cefazolin | ≤ 2/4/≥ 8 | ≤ 2/4/≥ 8 | ≤ 2/4/≥ 8 | ≤ 2/4/≥ 8 | ≤ 2/4/≥ 8 | ≤ 2/4/≥ 8 | ≤ 2/4/≥ 8 | ≤ 2/4/≥ 8 | - |
| Gentamicin | ≤ 4/8/≥ 16 | ≤ 4/8/≥ 16 | ≤ 4/8/≥ 16 | ≤ 4/8/≥ 16 | ≤ 4/8/≥ 16 | ≤ 4/8/≥ 16 | ≤ 4/8/≥ 16 | ≤ 4/8/≥ 16 | ? |
| Gamithromycin | - | - | - | - | - | - | - | - | ? |
| Trimethoprim-sulfamethoxazole | ≤ 2/-/≥ 4 | ≤ 2/-/≥ 4 | ≤ 2/-/≥ 4 | ≤ 2/-/≥ 4 | ≤ 2/-/≥ 4 | ≤ 2/-/≥ 4 | ≤ 2/-/≥ 4 | ≤ 2/-/≥ 4 | ? |
| Ceftiofur | ≤ 2/4/≥ 8 | ≤ 2/4/≥ 8 | ≤ 2/4/≥ 8 | ≤ 2/4/≥ 8 | ≤ 2/4/≥ 8 | ≤ 2/4/≥ 8 | ≤ 2/4/≥ 8 | - | ? |
| Chloramphenicol | ≤ 8/16/≥ 32 | ≤ 8/16/≥ 32 | ≤ 8/16/≥ 32 | ≤ 8/16/≥ 32 | ≤ 8/16/≥ 32 | ≤ 8/16/≥ 32 | ≤ 8/16/≥ 32 | ≤ 8/16/≥ 32 | - |
| Ceftazidime | ≤ 4/8/≥ 16 | ≤ 4/8/≥ 16 | ≤ 4/8/≥ 16 | ≤ 4/8/≥ 16 | ≤ 4/8/≥ 16 | ≤ 4/8/≥ 16 | ≤ 4/8/≥ 16 | - | - |
| Erythromycin | ? | ? | ? | ? | ? | ? | ? | ? | - |
| Clarithromycin | ? | ? | ? | ? | ? | ? | ? | - | - |
| Penicillin G | ? | ? | ? | ? | ? | ? | ? | ? | ? |
| Enrofloxacin | ? | ? | ? | ? | ? | ? | ? | ? | ≤ 0.25/-/≥ 2 |
| Imipenem | ≤ 1/2/≥ 4 | ≤ 1/2/≥ 4 | ≤ 1/2/≥ 4 | ≤ 1/2/≥ 4 | ≤ 1/2/≥ 4 | ≤ 1/2/≥ 4 | ≤ 1/2/≥ 4 | ≤ 1/2/≥ 4 | - |
| Doxycycline | ≤ 4/8/≥ 16 | ≤ 4/8/≥ 16 | ≤ 4/8/≥ 16 | ≤ 4/8/≥ 16 | ≤ 4/8/≥ 16 | ≤ 4/8/≥ 16 | ≤ 4/8/≥ 16 | ≤ 4/8/≥ 16 | - |
| Tetracycline | ≤ 4/8/≥ 16 | ≤ 4/8/≥ 16 | ≤ 4/8/≥ 16 | ≤ 4/8/≥ 16 | ≤ 4/8/≥ 16 | ≤ 4/8/≥ 16 | ≤ 4/8/≥ 16 | ≤ 4/8/≥ 16 | ≤ 2/-/≥ 8 |
| Ticarcillin | ≤ 16/32-64/≥ 128 | ≤ 16/32-64/≥ 128 | ≤ 16/32-64/≥ 128 | ≤ 16/32-64/≥ 128 | ≤ 16/32-64/≥ 128 | ≤ 16/32-64/≥ 128 | ≤ 16/32-64/≥ 128 | - | - |
| Ticarcillin-clavulanate | ? | ? | ? | ? | ? | ? | ? | - | - |
| Oxacillin | ? | ? | ? | ? | ? | ? | ? | ? | - |
| Rifampin | ? | ? | ? | ? | ? | ? | ? | ? | - |
| Sulfadimethoxine | - | - | - | - | - | - | - | - | ? |
| Tiamulin | - | - | - | - | - | - | - | - | ? |
| Tildipirosin | - | - | - | - | - | - | - | - | ? |
| Tilmicosin | - | - | - | - | - | - | - | - | ≤ 8/-/≥ 32 |
| Florfenicol | - | - | - | - | - | - | - | - | ≤ 16/-/≥ 64 |
| Tulathromycin | - | - | - | - | - | - | - | - | ? |
| Tylosin tartrate | - | - | - | - | - | - | - | - | ? |
| Neomycin | - | - | - | - | - | - | - | - | ? |
| Spectinomycin | - | - | - | - | - | - | - | - | ? |

^1^MIC values (µg/ml) presented in the table were obtained from the Sensititre Gram Negative Companion panel (COMPGN1F).

GREEN= no/few break points (isolate)

BLUE= no/few break points (antibiotic)

RED= resistant

DARK RED/WHITE TEXT= intrinsically resistant

YELLOW= highest test concentrations were lower than breakpoints

ORANGE= intermediate
